# Swimming-induced exercise promotes hypertrophy and vascularization of fast skeletal muscle fibres and activation of myogenic and angiogenic transcriptional programs in adult zebrafish

**DOI:** 10.1186/1471-2164-15-1136

**Published:** 2014-12-18

**Authors:** Arjan P Palstra, Mireia Rovira, David Rizo-Roca, Joan Ramon Torrella, Herman P Spaink, Josep V Planas

**Affiliations:** Departament de Fisiologia i Immunologia, Facultat de Biologia, Universitat de Barcelona, Barcelona, Spain; Institut de Biomedicina de la Universitat de Barcelona (IBUB), Barcelona, Spain; Institute for Marine Resources and Ecosystem Studies (IMARES), Wageningen Aquaculture, Wageningen UR, Yerseke, The Netherlands; Department of Molecular Cell Biology, Institute Biology, Leiden University, Leiden, The Netherlands

**Keywords:** Exercise, Swimming, Growth, Muscle, Transcriptome, Zebrafish

## Abstract

**Background:**

The adult skeletal muscle is a plastic tissue with a remarkable ability to adapt to different levels of activity by altering its excitability, its contractile and metabolic phenotype and its mass. We previously reported on the potential of adult zebrafish as a tractable experimental model for exercise physiology, established its optimal swimming speed and showed that swimming-induced contractile activity potentiated somatic growth. Given that the underlying exercise-induced transcriptional mechanisms regulating muscle mass in vertebrates are not fully understood, here we investigated the cellular and molecular adaptive mechanisms taking place in fast skeletal muscle of adult zebrafish in response to swimming.

**Results:**

Fish were trained at low swimming speed (0.1 m/s; non-exercised) or at their optimal swimming speed (0.4 m/s; exercised). A significant increase in fibre cross-sectional area (1.290 ± 88 vs. 1.665 ± 106 μm^2^) and vascularization (298 ± 23 vs. 458 ± 38 capillaries/mm^2^) was found in exercised over non-exercised fish. Gene expression profiling by microarray analysis evidenced the activation of a series of complex transcriptional networks of extracellular and intracellular signaling molecules and pathways involved in the regulation of muscle mass (e.g. IGF-1/PI3K/mTOR, BMP, MSTN), myogenesis and satellite cell activation (e.g. PAX3, FGF, Notch, Wnt, MEF2, Hh, EphrinB2) and angiogenesis (e.g. VEGF, HIF, Notch, EphrinB2, KLF2), some of which had not been previously associated with exercise-induced contractile activity.

**Conclusions:**

The results from the present study show that exercise-induced contractile activity in adult zebrafish promotes a coordinated adaptive response in fast muscle that leads to increased muscle mass by hypertrophy and increased vascularization by angiogenesis. We propose that these phenotypic adaptations are the result of extensive transcriptional changes induced by exercise. Analysis of the transcriptional networks that are activated in response to exercise in the adult zebrafish fast muscle resulted in the identification of key signaling pathways and factors for the regulation of skeletal muscle mass, myogenesis and angiogenesis that have been remarkably conserved during evolution from fish to mammals. These results further support the validity of the adult zebrafish as an exercise model to decipher the complex molecular and cellular mechanisms governing skeletal muscle mass and function in vertebrates.

**Electronic supplementary material:**

The online version of this article (doi:10.1186/1471-2164-15-1136) contains supplementary material, which is available to authorized users.

## Background

In all animals, skeletal muscle has evolved to play a fundamental role in locomotion and energy metabolism. The adult skeletal muscle is a post-mitotic tissue with unique plasticity, that is, it has an extraordinary ability to adjust to changes in its physiological environment by altering its excitability, its contractile and metabolic phenotype and its mass. Importantly, skeletal muscle usage is able to exert profound changes in its phenotype. The induction of contractile activity by exercise represents a physiological stimulus that elicits important adaptive responses in skeletal muscle either directly by mechanical strain or indirectly through its ability to increase intracellular calcium levels in response to neural stimulation [1–3]. These adaptive responses, that ultimately serve to increase fitness, are governed by genetic programs involving complex transcriptional responses that depend on the activity of transcription factors and chromatin modifying enzymes [4, 5] and are not fully understood, even in mammals. Due to the known beneficial effects of exercise-induced skeletal muscle activity for preventing cardiovascular (e.g. coronary heart disease, hypertension), metabolic (e.g. type 2 diabetes mellitus, obesity) and age-related (e.g. sarcopenia) conditions [6, 7] in humans, knowledge on the pathways that participate in the adaptation of skeletal muscle to exercise-induced activity is of crucial importance for understanding the basic mechanisms involved in this process. This may also be important for assessing possible modulatory effects of exercise on muscle regeneration and for identifying potential pharmaceutical targets useful for the treatment of muscle disorders.

After two decades as a research model, the zebrafish (*Danio rerio*) has made important contributions to our current knowledge on skeletal muscle developmental biology [8, 9] and the pathological basis of neuromuscular disorders, such as muscular dystrophy and myopathies [10, 11]. This has been possible because the zebrafish skeletal muscle has many molecular features (i.e. a conserved transcriptional network regulating myogenesis), as well as histological and ultrastructural features, that are very similar to those in the mammalian muscle [12, 13]. Furthermore, the zebrafish has anatomically separated fast- and slow-twitch fibres as a result of distinct ontogenic programs making this an interesting model to investigate fibre type specification [9] and fibre growth [14, 15]. Therefore, the zebrafish, due its tractability and the ease of genetic manipulation coupled with the vast genetic and genomic tools available, has tremendous potential to contribute importantly to our knowledge on skeletal muscle function and, specifically, on the mechanisms responsible for the regulation of adult muscle mass in vertebrates, including humans. However, most of the current knowledge on the regulation of skeletal muscle mass in zebrafish is derived from studies on the effects of muscle inactivity or injury and on genetic models of human muscle disorders [10, 14, 16] and not based on models of increased skeletal muscle activity, such as induced by exercise. In order to begin to elucidate the effects of exercise-induced contractile activity on skeletal muscle physiology in adult zebrafish and to contribute to its establishment as an exercise model species in fish and biomedical research, we recently studied the swimming economy in adult zebrafish and established its optimal swimming speed (i.e. the swimming speed at which the cost of transport is lowest and the energetic efficiency is highest) [17]. By applying these aerobic exercise conditions in a swimming training protocol for 20 days, a significant exercise-induced growth was demonstrated for the first time in adult zebrafish that was associated with the regulated expression of growth marker genes in fast muscle [17]. Based on the results from that study, we put forward the notion that zebrafish can be used as an exercise model for studying muscle growth. Therefore, the present study aimed to describe the cellular and molecular adaptive response of fast skeletal muscle to swimming-induced exercise in adult zebrafish and further validate the zebrafish as a useful animal model for investigating the effects of exercise on skeletal muscle physiology in vertebrates.

In the present study, we report on the effects of exercise training on the cellular and molecular characteristics of fast muscle in adult zebrafish. Our results indicate that exercise-induced contractile activity in adult zebrafish promotes a coordinated adaptive response in fast muscle that leads to increased muscle mass by hypertrophy and increased vascularization by angiogenesis. These phenotypic changes are likely the result of the transcriptional activation of a series of complex networks of extracellular and intracellular signaling molecules and pathways involved in the regulation of muscle mass, myogenesis and angiogenesis in adult zebrafish, some not previously associated with exercise-induced contractile activity. Moreover, the present study reinforces the notion that zebrafish is a valid and promising animal model to promote our understanding of the complex mechanisms responsible for the regulation of adult skeletal muscle mass by exercise.

## Results

### Exercise training promotes changes in fibre morphometry and capillarization in fast muscle of adult zebrafish

**Figure 1:**
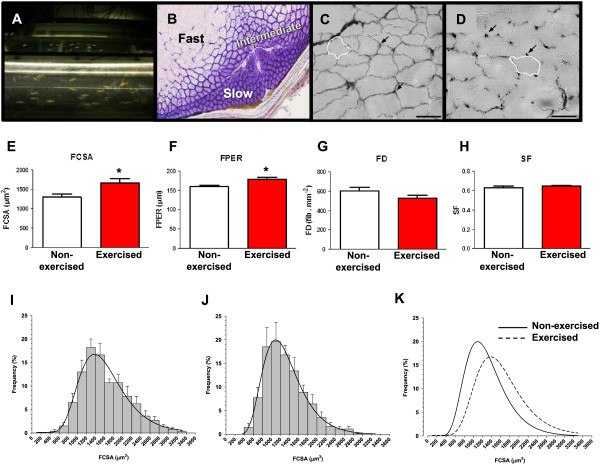
**Morphometrical fibre parameters in fast muscle of exercised and non-exercised adult zebrafish. A**: Image of the swim tunnels used for exercise training. Front tunnel: exercised zebrafish; back tunnel: non-exercised zebrafish. **B-D**: Images of zebrafish cross-sectional white muscle. Images correspond to representative serial transverse secions stained **(B)** for succinate dehydrogenase for the identification of fast, intermediate (pink) and slow muscle fibres; **(C and D)** for ATPase for capillary demonstration (arrows) and FCSA and FPER measures (white drawing) from a non-exercised **(C)** and an exercised **(D)** adult zebrafish. Bar represents 50 μm. Morphometric fibre parameters measured in non-exercised and exercised zebrafish were: *FCSA*, fibre cross-sectional area (μm^2^) **(E)**; *FPER*, fibre perimeter (μm) **(F)**; *FD*, fibre density (fibres/mm^2^) **(G)**; *SF*, shape factor **(H)**. Statistical significance values between non-exercised and exercised zebrafish: *P < 0.05. Values are mean ± SEM from a sample size of n = 8 for each condition. I-J: Fibre cross-sectional area histograms from fast muscle of exercised **(I)** and non-exercised **(J)** adult zebrafish. In K, the two overlapped curves are shown. Muscle fibre areas were grouped in intervals of 200 μm^2^ and the data correspond to mean ± SEM frequency of six animals. Curves represent a log-normal regression of four parameters. Regression parameters are shown in Additional file 1. See Methods for details.

**Figure 2:**
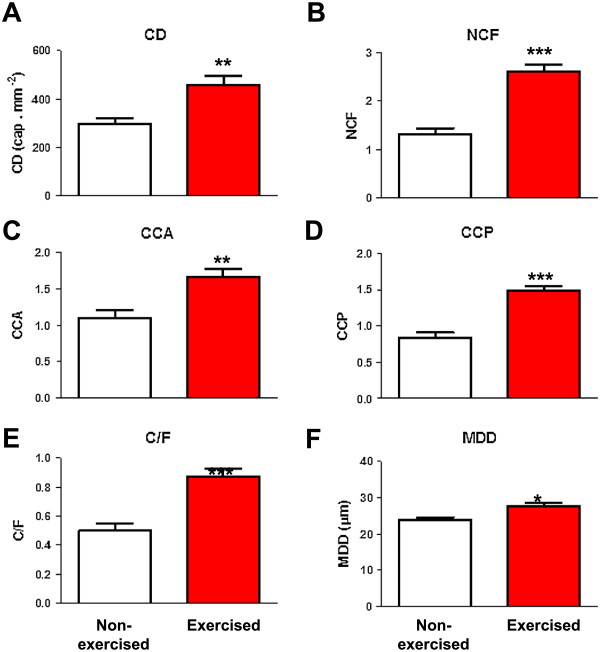
**Morphometrical capillarity parameters in fast muscle of exercised and non-exercised adult zebrafish.** Parameters measured were: *CD*, capillary density (capillaries/mm^2^) **(A)**; *NCF*, number of capillaries in contact with each fibre **(B)**; *CCA*, relationship between NCF and the FCSA (NCF · 10^3^/FCSA) **(C)**; *CCP*, relationship between NCF and the FPER (NCF · 10^2^/FPER) **(D)**; *C/F*, capillary-to-fibre ratio (CD/FD) **(E)** and *MDD*, maximal diffusion distance between the capillary and the centre of the fibre **(F)**. Statistical significance values between non-exercised and exercised zebrafish: *P < 0.05, **P < 0.01, ***P < 0.001. Values are mean ± SEM from a sample size of n = 8 for each condition.

Morphometrical assessment of fast muscle in exercised and non-exercised adult zebrafish was performed to evaluate the effects of exercise training on skeletal muscle cellular characteristics (Figure 1A-D). Exercised zebrafish showed a significant (P < 0.05) increase (29%) in fibre cross-sectional area (FCSA) (Figure 1E). Furthermore, exercised zebrafish also showed a significant (P < 0.05) increase in fibre perimeter (12%) (Figure 1F) and a non-significant decrease in fibre density (Figure 1G) in fast muscle without changes in the shape of the fibres, as indicated by the absence of differences in fibre circularity (shape factor) between exercised and non-exercised zebrafish (Figure 1H). Fast muscle fibre frequency distribution analyses in non-exercised and exercised zebrafish evidenced that log-normal regression curves were centered around higher FCSA values in exercised (approximately 1.400 μm^2^) (Figure 1I) over non-exercised zebrafish (approximately 1.100 μm^2^) (Figure 1J), as also deduced by the significant (P < 0.0001) shift to the right of the regression curve of exercised zebrafish relative to that of non-exercised zebrafish (Figure 1K; Additional file 1: Table S1). When the mean percentages of muscle fibres were grouped into three major intervals of FCSA and quantified, exercised zebrafish presented significantly lower percentages of small fibres (FCSA < 1.200 μm^2^) but significantly higher percentages of medium (with sizes between 1.200 μm^2^ and 2.400 μm^2^) and large fibres (FCSA > 2.400 μm^2^) than non-exercised zebrafish (Additional file 1: Table S1). Therefore, these observations clearly indicate that fibre size was significantly increased in exercised zebrafish and, consequently, that exercise training caused hypertrophy of fast muscle fibres in adult zebrafish.Exercise training also induced vascularization of the fast muscle in zebrafish, as assessed by histochemical quantification of capillaries (Figure 1C,D). The total capillary density increased by 54% (P < 0.01) in fast muscle of exercised relative to non-exercised zebrafish (Figure 2A). Importantly, exercise training caused a significant (P < 0.001) increase in the number of capillaries in contact with each fibre (98%) (Figure 2B) as well as a significantly greater number of capillaries per fibre area (52%) (Figure 2C) and per fibre perimeter (76%) (Figure 2D) in fast muscle of adult zebrafish. The capillary-to-fibre ratio (CD/FD) increased by 74% (P < 0.001) in exercised zebrafish (Figure 2E). However, maximal diffusion distance between the capillary and the centre of the fibre was modestly but significantly (P < 0.05) increased (15%) in the fast muscle of exercised zebrafish (Figure 2F), likely as a result of a greater fibre size.

### Exercise training induces profound transcriptomic changes in fast muscle of adult zebrafish

In order to gain insight into the molecular basis of the increase in fast muscle fibre hypertrophy and vascularization in exercised adult zebrafish, we evaluated the transcriptomic response of fast muscle to swimming-induced exercise by microarray analysis. Gene expression profiling of the zebrafish fast muscle evidenced important transcriptomic changes, with 1.625 genes down-regulated and 2.851 genes up-regulated in response to exercise training. Initial classification of differentially expressed genes by Gene Ontology categories using DAVID revealed a significant (p < 0.05) enrichment in functional categories related to muscle development and differentiation, sarcomeric contractile elements, cell cycle and apoptosis, protein, carbohydrate and lipid metabolism, oxidative phosphorylation and blood vessel development (Table 1). Importantly, exercise training modulated the expression of genes involved in a wide variety of processes that are responsible for the functional contractile activation of skeletal muscle fibres: activation of neuromuscular communication (e.g. *ache, chrm2, scn4b*), translation of nerve-evoked electrical activity into an intracellular Ca^2+^ signal (i.e. excitation-contraction coupling) (e.g. *atp2a1, calm1, casq1, pvalb, ppp3ca, ryr1*), sarcomere contraction (e.g. *actn4, actb, actc1, capzb, mybph, myh11, myl2, myl9, mylpf, tpm1, tnni2, tnnt3, ttn*), cytoskeletal transmission of sarcomeric contractile force to the sarcolemma (e.g. *ank2, dag1, des, dmd, dtnbp1, flnc, itga2b, itgb4, lmna, myoz1, myoz2, sntb1, sptbn, vim*) and force transmission and muscle structure maintenance by the extracellular matrix (e.g. *col1a1, col8a2, col16a1, lama1, lamc3, loxl2, loxl3, sdcbp, tnc*) (Table 2). Furthermore, exercise training also altered the expression of fast muscle genes involved in the control of muscle growth and development, such as growth factors (e.g. *egfr, fgf13, fgf18, fgf20, fgfr1, fgfr2, fst, igf1r, igfbp1, igfbp3, igfbp7, igf2, mstn, ngf, tgfb1, tgfb2*), extracellular signaling molecules (e.g. *bmp1, bmp4, bmpr1a, bmpr1b, ihh, nog, shh, wnt7a, wnt10a*), components of intracellular signaling pathways (e.g. *esrra, esrrb, esrrg, foxa1, foxo3, irs1, irs2, mapk1, mapk8, mapk13, mapk14pik3c2b, smad6*) and transcriptional regulators of myogenesis (e.g. *hdac4, hadc6, id1, id3, mef2a, mef2ca, mef2d, pax3*) (Table 2).

**Table 1:** **Functional annotation analysis based on GO terms in zebrafish fast muscle in response to swimming (DAVID)**

GO Term		Count	P-value
GO:0014706	Striated muscle tissue development	41	0,0014644
GO:0051146	Striated muscle cell differentiation	31	0,0042799
GO:0030239	Myofibril assembly	10	0,0227268
GO:0031032	Actomyosin structure organization	14	0,0028624
GO:0032956	Regulation of actin cytoskeleton organization	28	0,0318891
GO:0040007	Growth	65	1,72E-05
GO:0045926	Negative regulation of growth	36	0,0074826
GO:0000278	Mitotic cell cycle	124	8,01E-08
GO:0051726	Regulation of cell cycle	92	0,0053137
GO:0006915	Apoptosis	161	0,0014411
GO:0043065	Positive regulation of apoptosis	110	0,0295542
GO:0006457	Protein folding	60	1,67E-04
GO:0030162	Regulation of proteolysis	19	0,0245616
GO:0006468	Protein amino acid phosphorylation	173	0,0038954
GO:0006511	Ubiquitin-dependent protein catabolic process	84	2,55E-06
GO:0006979	Response to oxidative stress	46	0,0414755
GO:0080135	Regulation of cellular response to stress	31	0,0418028
GO:0045454	Cell redox homeostasis	21	0,0378637
GO:0015980	Energy derivation by oxidation of organic compounds	51	1,68E-04
GO:0022900	Electron transport chain	41	5,67E-04
GO:0006754	ATP biosynthetic process	32	0,0025614
GO:0006119	Oxidative phosphorylation	44	5,45E-07
GO:0044262	Cellular carbohydrate metabolic process	111	6,80E-04
GO:0006096	Glycolysis	21	9,35E-04
GO:0044255	Cellular lipid metabolic process	157	4,84E-06
GO:0006635	Fatty acid beta-oxidation	15	7,99E-04
GO:0006631	Fatty acid metabolic process	75	2,13E-07
GO:0006633	Fatty acid biosynthetic process	30	0,0013958
GO:0006520	Cellular amino acid metabolic process	71	5,16E-04
GO:0042180	Cellular ketone metabolic process	190	1,95E-11
GO:0001568	Blood vessel development	78	1,68E-04
GO:0048514	Blood vessel morphogenesis	68	3,13E-04
GO:0001570	Vasculogenesis	16	0,0194009
GO:0045449	Regulation of transcription	593	0,0425425
GO:0043408	Regulation of MAPKKK cascade	35	0,011466
GO:0051101	Regulation of DNA binding	42	0,0010788
GO:0007243	Protein kinase cascade	95	0,0387284
GO:0030509	BMP signaling pathway	17	0,0170619
GO:0016055	Wnt receptor signaling pathway	53	2,84E-06

**Table 2:** **Selected differentially expressed genes in fast muscle of exercised zebrafish that participate in the contractile activation of skeletal muscle fibers**

Gene name gene description		FC	Gene name gene description		FC
Muscle contraction			Muscle growth and development		
*capn8*	Calpain 8	**4.11**	*fgf20*	Fibroblast growth factor 20	**8.94**
*actn4*	Actinin, alpha 4	**3.99**	*hdac6*	Histone deacetylase 6	**6.42**
*myh11*	Myosin, heavy chain 11, smooth muscle	**3.63**	*fgf18*	Fibroblast growth factor 18	**6.30**
*camk2n2*	Ca/calmodulin-dependent protein kinase II inhibitor 2	**3.38**	*wnt10a*	Wingless-type MMTV integration site, 10A	**6.25**
*pvalb*	Parvalbumin	**3.24**	*pax3*	Paired box 3	**6.21**
*tnni2*	Troponin I type 2 (skeletal, fast)	**3.12**	*tgfb2*	Transforming growth factor, beta 2	**5.35**
*capn3*	Calpain 3, (p94)	**3.08**	*nog*	Noggin	**4.90**
*nfatc4*	Nuclear factor of activated T-cells, calcineurin-dep. 4	**3.08**	*esrra*	Estrogen-related receptor alpha	**4.73**
*capn2*	Calpain 2, (m/II) large subunit	**2.92**	*wnt7a*	Wingless-type MMTV integration site, 7A	**4.60**
*tmod4*	Tropomodulin 4 (muscle)	**2.79**	*mstn*	Myostatin	**4.41**
*nfatc1*	Nuclear factor of activated T-cells, , calcineurin-dep. 1	**2.76**	*foxa1*	Forkhead box A1	**4.22**
*capzb*	Capping protein (actin filament) muscle Z-line, beta	**2.75**	*fgfr2*	Fibroblast growth factor receptor 2	**4.05**
*casq1*	Calsequestrin 1 (fast-twitch, skeletal muscle)	**2.68**	*shh*	Sonic hedgehog	**3.78**
*myl2*	Myosin, light chain 2, regulatory, cardiac, slow	**2.64**	*fzd2*	Frizzled family receptor 2	**3.08**
*ppp3cc*	Protein phosphatase 3, catalytic subunit, gamma	**2.61**	*pik3c2b*	Phosphatidylinositol-4-p- 3-kinase c2b	**3.06**
*capn5*	Calpain 5	**2.60**	*fgf13*	Fibroblast growth factor 13	**3.01**
*ttn*	Titin	**2.58**	*mapk1*	Mitogen-activated protein kinase 1	**3.00**
*ppp3ca*	Protein phosphatase 3, catalytic subunit, alpha isozyme	**2.52**	*fzd10*	Frizzled family receptor 10	**2.94**
*mylpf*	Myosin light chain, phosphorylatable, fast skel. muscle	**2.26**	*ihh*	Indian hedgehog	**2.91**
*mybph*	Myosin binding protein H	**2.17**	*fzd8*	Frizzled family receptor 8	**2.87**
*capn10*	Calpain 10	**2.15**	*esrrb*	Estrogen-related receptor beta	**2.61**
*cacna1s*	Calcium channel, voltage-dependent, L type, alpha 1S	**2.11**	*bmpr1a*	Bone morphogenetic protein receptor, IA	**2.58**
*camk2a*	Calcium/calmodulin-dependent protein kinase II alpha	**1.98**	*ngf*	Nerve growth factor (beta polypeptide)	**2.55**
*camk2d*	Calcium/calmodulin-dependent protein kinase II delta	**1.97**	*igf1r*	Insulin-like growth factor 1 receptor	**2.53**
*nfatc3*	Nuclear factor of activated T-cells, calcineurin-dep. 3	**1.92**	*bmp1*	Bone morphogenetic protein 1	**2.46**
*acta2*	Actin, alpha 2, smooth muscle, aorta	**1.92**	*dvl1*	Dishevelled, dsh homolog 1 (Drosophila)	**2.43**
*mylk*	Myosin light chain kinase	**1.87**	*smad2*	SMAD family member 2	**2.40**
*tpm4*	Tropomyosin 4	**1.78**	*bmp4*	Bone morphogenetic protein 4	**2.38**
*myl9*	Myosin, light chain 9, regulatory	**1.77**	*igfbp7*	Insulin-like growth factor binding protein 7	**2.36**
*ryr1*	Ryanodine receptor 1 (skeletal)	**1.77**	*esrrg*	Estrogen-related receptor gamma	**2.36**
*tpm1*	Tropomyosin 1 (alpha)	**1.71**	*bmpr1b*	Bone morphogenetic protein receptor, IB	**2.27**
*atp2a1*	ATPase, Ca transporting, cardiac muscle, fast twitch 1	**1.70**	*erbb2*	v-erb-b2 erythroblastic leukemia. 2	**2.27**
*actc1*	Actin, alpha, cardiac muscle 1	**1.68**	*mapk13*	Mitogen-activated protein kinase 13	**2.23**
*cacng1*	Calcium channel, voltage-dependent, gamma subunit 1	**1.61**	*fst*	Follistatin	**2.17**
*myl12b*	Myosin, light chain 12B, regulatory	**−1.59**	*mapk8*	Mitogen-activated protein kinase 8	**2.12**
*s100a4*	S100 calcium binding protein A4	**−1.63**	*smad6*	SMAD family member 6	**2.06**
*calm1*	Calmodulin 1 (phosphorylase kinase, delta)	**−2.04**	*fgfr1*	Fibroblast growth factor receptor 1	**1.96**
*actg2*	Actin, gamma 2, smooth muscle, enteric	**−3.87**	*irs2*	Insulin receptor substrate 2	**1.91**
*tnnt3*	Troponin T type 3 (skeletal, fast)	**−7.01**	*runx2*	Runt-related transcription factor 2	**1.90**
**Cytoskeleton**			*igfbp1*	Insulin-like growth factor binding protein 1	**1,89**
*ank2*	Ankyrin 2, neuronal	**11.01**	*irs1*	Insulin receptor substrate 1	**1.78**
*plec*	Plectin	**3.31**	*acvr2b*	Activin A receptor, type IIB	**1.74**
*myoz1*	Myozenin 1	**2.41**	*tgfb1*	Transforming growth factor, beta 1	**1.71**
*myoz2*	Myozenin 2	**2.28**	*mef2d*	Myocyte enhancer factor 2D	**1.71**
*dag1*	Dystroglycan 1 (dystrophin-associated glycoprotein 1)	**2.26**	*hdac4*	Histone deacetylase 4	**1.71**
*itgb4*	Integrin, beta 4	**1.99**	*igfbp3*	Insulin-like growth factor binding protein 3	**1.66**
*itga2b*	Integrin, alpha 2b	**1.95**	*mef2a*	Myocyte enhancer factor 2A	**1.66**
*dmd*	Dystrophin	**1.94**	*igf2*	Insulin-like growth factor 2	**1.61**
*filip1*	Filamin A interacting protein 1	**1.88**	*pten*	Phosphatase and tensin homolog	**−1.54**
*sntb1*	Syntrophin, beta 1 (dystrophin-associated protein A1)	**1.78**	*mef2c*	Myocyte enhancer factor 2C	**−1.59**
*vim*	Vimentin	**1.61**	*egfr*	Epidermal growth factor receptor	**−2.13**
*lmna*	Lamin A/C	**−1.51**	*id3*	Inhibitor of DNA binding 3	**−2.39**
*dtnbp1*	Dystrobrevin binding protein 1	**−1.76**	*srf*	Serum response factor	**−2.68**
*flnc*	Filamin C, gamma	**−1.88**	*mapk14*	Mitogen-activated protein kinase 14	**−2.78**
**Neuromuscular junction**			**Extracelular matrix**		
*ache*	Acetylcholinesterase	**8.86**	*col8a2*	Collagen, type VIII, alpha 2	**12.06**
*vamp1*	Vesicle-associated membrane prot. 1 (synaptobrevin1)	**3.78**	*lamc3*	Laminin, gamma 3	**10.06**
*chrm2*	Cholinergic receptor, muscarinic 2	**3.65**	*col16a1*	Collagen, type XVI, alpha 1	**6.17**
*snap25*	Synaptosomal-associated protein, 25kDa	**3.08**	*col1a2*	Collagen, type I, alpha 2	**3.12**
*scn4b*	Sodium channel, voltage-gated, type IV, beta subunit	**3.01**	*bgn*	Biglycan	**2.98**
*syn2*	Synapsin II	**2.70**	*loxl2*	Lysyl oxidase-like 2	**2.92**
*syt1*	Synaptotagmin I	**2.22**	*mmp14*	Matrix metallopeptidase 14	**2.69**
*rims2*	Regulating synaptic membrane exocytosis 2	**1.93**	*tnc*	Tenascin C	**2.53**
*scnm1*	Sodium channel modifier 1	**1.65**	*mmp10*	Matrix metallopeptidase 10 (stromelysin 2)	**2.06**
*syncrip*	Synaptotagmin binding, cytoplasmic RNA interact. pro.	**−1.54**	*sdcbp*	Syndecan binding protein (syntenin)	**−2.26**

Data are shown as fold change (FC).

Functional categories are indicated in bold.

Consistent with the increased vascularization of fast muscle by exercise training, the expression of a number of genes involved in angiogenesis was altered in fast muscle, including angiopoietins (e.g. *angpt2, angptl2, angptl3*), members of the ephrin family and receptors (e.g. *efna2, efna3, efnb2, efnb3, epha4, epha7, ephb4*), members of the notch family (e.g. *dll1, jag1, jag2, notch1, notch2*), hypoxia-inducible factors (e.g. *hif1an, hif3a*), *gata1* and *nrp1* (Table 3). Among genes involved in metabolism with altered mRNA expression levels in fast muscle of exercised zebrafish were genes responsible for the metabolic provision of ATP in skeletal muscle such as *pdha1*, members of the ATP-phosphagen system (e.g. *ak1, ak2, a3, ckm, ckmt2*), and multiple components of the mitochondrial electron transport chain (e.g. *ndufa, cox, atp5*) and the tricarboxylic acid (TCA) cycle (e.g. *fh, idh3b, idh3g, mdh1, mdh2, ogdh, sdha*) (Table 3). Other differentially expressed genes included genes known to participate in energy metabolism (e.g. *adipor2*, *mb*, *prkaa1*, *prkab1*, *prkag1*, *ppara*, *ppard*, *ucp2* and *ucp3*). Moreover, genes involved in the metabolic utilization of energy substrates as fuel, namely lipids (e.g. *cpt2, capt1a, fabp3, lpl, mcat, slc27a2*) and carbohydrates (e.g. *aldoa, aldoc, eno1, gapdh, g6pc, gpi, hk2, pfkm, pgk1, pkm*), also showed altered expression in fast muscle of exercised zebrafish. Importantly, exercise training altered the expression of genes involved in protein synthesis and degradation in fast muscle (e.g. *eif4e, eif4ebp1, fbxo32, foxo3, pdk1, pdk2, rps6ka1, trim63*). Finally, exercise training caused alterations in the expression of immune-related genes (e.g. *il11ra*, *il12b*, *il13ra2*, *il17d*, *il17dr*, *il20*, *il20ra*, *irf3*, *mif*, *mst1* and *traf6*) in fast muscle of adult zebrafish (Table 3).

**Table 3:** **Selected differentially expressed genes in fast muscle of exercised adult zebrafish that participate in angiogenesis, immune-related processess and metabolism**

Gene name gene description		FC	Gene name gene description		FC
Angiogenesis			Energy metabolism		
*klf2*	Kruppel-like factor 2 (lung)	**8.52**	*cpt1a*	Carnitine palmitoyltransferase 1A (liver)	**5.23**
*robo2*	Roundabout, axon guidance receptor, homolog 2 (Drosophila)	**4.30**	*pfkm*	Phosphofructokinase, muscle	**3.84**
*angpt2*	Angiopoietin 2	**3.90**	*prkaaq*	Protein kinase, AMP-activated, alpha 1 cat.	**3.66**
*angptl3*	Angiopoietin-like 3	**3.47**	*elovl4*	ELOVL fatty acid elongase 4	**3.65**
*efna3*	Ephrin-A3	**3.45**	*prkag1*	Protein kinase, AMP-activated, gamma 1 catalytic subunit	**3.50**
*gata1*	GATA binding protein 1 (globin transcription factor 1)	**3.00**	*acadl*	Acyl-CoA dehydrogenase, long chain	**3.20**
*epha4*	EPH receptor A4	**2.96**	*ppard*	Peroxisome proliferator-activated receptor d	**3.19**
*nrp1*	Neuropilin 1	**2.89**	*aldoa*	Aldolase A, fructose-bisphosphate	**3.18**
*mmp14*	Matrix metallopeptidase 14 (membrane-inserted)	**2.69**	*mcat*	Malonyl CoA:ACP acyltransferase (mitochondrial)	**3.14**
*nos1*	Nitric oxide synthase 1 (neuronal)	**2.65**	*slc27a2*	Solute carrier family 27 (fatty acid transporter), member 2	**3.10**
*notch1*	Notch 1	**2.60**	*prkab1*	Protein kinase, AMP-activated, beta 1 non-catalytic subunit	**3.06**
*sema3f*	Sema domain, immunoglobulin domain (Ig), short basic domain, secreted, (semaphorin) 3F	**2.57**	*mb*	Myoglobin	**2.95**
*slit3*	Slit homolog 3 (Drosophila)	**2.53**	*cox7c*	Cytochrome c oxidase subunit VIIc	**2.85**
*amot*	Angiomotin	**2.34**	*ppara*	Peroxisome proliferator-activated receptor alpha	**2.77**
*hey2*	Hairy/enhancer-of-split related with YRPW motif 2	**2.20**	*tfb2m*	Transcription factor B2, mitochondrial	**2.42**
*tp63*	Tumor protein p63	**2.16**	*fbp1*	Fructose-1,6-bisphosphatase 1	**2.39**
*mmp10*	Matrix metallopeptidase 10 (stromelysin 2)	**2.06**	*g6pc*	Glucose-6-phosphatase, catalytic subunit	**2.38**
*s1pr1*	Sphingosine-1-phosphate receptor 1	**2.03**	*pdha1*	Pyruvate dehydrogenase (lipoamide) alpha 1	**2.24**
*ephb4*	EPH receptor B4	**1.97**	*ckm*	Creatine kinase, muscle	**2.24**
*nr2f2*	Nuclear receptor subfamily 2, group F, member 2	**1.95**	*fh*	Fumarate hydratase	**2.20**
*efnb3*	Ephrin-B3	**1.94**	*ogdh*	Oxoglutarate hydrogenase (lipoamide)	**2.19**
*hif3a*	Hypoxia inducible factor 3, alpha subunit	**1.92**	*gapdh*	Glyceraldehyde-3-phosphate dehydrogenase	**2.19**
*epha7*	EPH receptor A7	**1.91**	*adh5*	Alcohol dehydrogenase 5 (class III)	**2.18**
*angptl2*	Angiopoietin-like 2	**1.90**	*cox5a*	Cytochrome c oxidase subunit Va	**2.12**
*nos2*	Nitric oxide synthase 2, inducible	**1.85**	*pgk1*	Phosphoglycerate kinase 1	**2.02**
*cdc42ep2*	CDC42 effector protein (Rho GTPase binding) 2	**1.83**	*fads6*	Fatty acid desaturase 6	**1.99**
*efna2*	Ephrin-A2	**1.83**	*mdh2*	Malate dehydrogenase 2, NAD (mitochondrial)	**1.97**
*nr2f1*	Nuclear receptor subfamily 2, group F, member 1	**1.83**	*cox6a2*	Cytochrome c oxidase subunit VIa polypeptide 2	**1.97**
*jag1*	Jagged 1	**1.80**	*ndufv1*	NADH dehydrogenase (ubiquinone) flavoprotein 1, 51kDa	**1.94**
*slit2*	Slit homolog 2 (Drosophila)	**1.79**	*fabp3*	Fatty acid binding protein 3, muscle and heart	**1.94**
*hey1*	Hairy/enhancer-of-split related with YRPW motif 1	**1.78**	*slcad*	Solute carrier family 2 (facilitated glucose transporter), member 2	**1.92**
*hif1an*	Hypoxia inducible factor 1, alpha subunit inhibitor	**1.73**	*atp5h*	ATP synthase, H+ transporting, mitochondrial Fo complex, subunit d	**1.91**
*foxc1*	Forkhead box C1	**1.68**	*ucp3*	Uncoupling protein 3 (mitochondrial )	**1.88**
*efnb2*	Ephrin-B2	**1.63**	*cpt2*	Carnitine palmitoyltransferase 2	**1.86**
*jag2*	Jagged 2	**1.54**	*ndufb1*	NADH dehydrogenase (ubiquinone) 1 beta subcomplex, 1, 7kDa	**1.82**
*vegfc*	Vascular endothelial growth factor C	**1.36**	*ckmt2*	Creatine kinase, mitochondrial 2 (sarcomeric)	**1.82**
*dll1*	Delta-like 1 (Drosophila)	**−1.25**	*mdh1*	Malate dehydrogenase 1, NAD (soluble)	**1.77**
*rac1*	Ras-related C3 botulinum toxin substrate 1	**−1.57**	*sdha*	Succinate dehydrogenase complex, subunit A,	**1.74**
*rock2*	Rho-associated, coiled-coil containing protein kinase 2	**−1.61**	*mt-atp6*	ATP synthase F0 subunit 6	**1.70**
*notch2*	Notch 2	**−1.89**	*acacb*	Acetyl-CoA carboxylase beta	**1.70**
*cdc42*	Cell division cycle 42	**−1.97**	*ucp2*	Uncoupling protein 2 (mitochondrial)	**1.68**
*aggf1*	Angiogenic factor with G patch and FHA domains 1	**−2.10**	*atp5o*	ATP synthase, H+ transporting, mitochondrial F1 complex, O subunit	**1.68**
**Immune-related factors**			*eno1*	Enolase 1, (alpha)	**1,68**
*traf6*	TNF receptor-associated factor 6, E3 ubiquitin protein ligase	**10.78**	*cox4i1*	Cytochrome c oxidase subunit IV isoform 1	**1.68**
*il17D*	Interleukin 17D	**6.51**	*cox7a2l*	Cytochrome c oxidase subunit VIIa polypeptide 2 like	**1.67**
*ptgs1*	Prostaglandin-endoperoxide synthase 1	**5.81**	*atp5f1*	ATP synthase, H+ transporting, mitochondrial Fo complex, subunit B1	**1.65**
*irak1bp1*	Interleukin-1 receptor-associated kinase 1 BP 1	**4.98**	*nrf1*	Nuclear respiratory factor 1	**1.62**
*irf3*	Interferon regulatory factor 3	**4.60**	*ldhb*	Lactate dehydrogenase B	**1.60**
*il29ra*	Interleukin 20 receptor, alpha	**3.91**	*adipor2*	Adiponectin receptor 2	**1.56**
*il12b*	Interleukin 12B	**3.68**	*lpl*	Lipoprotein lipase	**−1.51**
*il11ra*	Interleukin 11 receptor, alpha	**3.24**	*eifsb4*	Eukaryotic translation initiation factor 2B, subunit 4 delta, 67kDa	**−1.58**
*ptgr1*	Prostaglandin reductase 1	**3.21**	*gpi*	Glucose-6-phosphate isomerase	**−1.76**
*ptgds*	Prostaglandin D2 synthase 21kDa (brain)	**3.18**	*ndufaf4*	NADH dehydrogenase (ubiquinone) complex I, assembly factor 4	**−1.88**
*ptgis*	Prostaglandin I2 (prostacyclin) synthase	**2.87**	*aldoc*	Aldolase C, fructose-bisphosphate	**−2.03**
*il13ra2*	Interleukin 13 receptor, alpha 2	**2.54**	*pkm*	Pyruvate kinase, muscle	**−2.24**
*il20*	Interleukin 20	**2.44**	*hk2*	Hexokinase 2	**−2.36**
*tnfrsf19*	Tumor necrosis factor receptor superfamily, member 19	**2.43**	**Protein synthesis and degradation**		
*nkrf*	NFKB repressing factor	**1.77**	*pdk2*	Pyruvate dehydrogenase kinase, isozyme 2	**6.87**
*il17rd*	Interleukin 17 receptor D	**1.74**	*fbxo32*	F-box protein 32	**6.01**
*mst1*	Macrophage stimulating 1 (hepatocyte growth factor-like)	**1.66**	*pdk1*	Pyruvate dehydrogenase kinase, isozyme 1	**2.19**
*mif*	Macrophage migration inhibitory factor (glycosylation-inhibiting factor)	**−1.50**	*foxo3*	Forkhead box O3	**2.08**
*ilf3*	Interleukin enhancer binding factor 3, 90kDa	**−1.77**	*trim63*	Tripartite motif containing 63, E3 ubiquitin protein ligase	**2.02**
*ptges3*	Prostaglandin E synthase 3 (cytosolic)	**−2.29**	*rps6ka1*	Ribosomal protein S6 kinase, 90kDa, polypeptide 1	**1.96**
*il21r*	Interleukin 21 receptor	**−2,59**	*eif4e*	Eukaryotic translation initiation factor 4E	**−1,89**
*irak4*	Interleukin-1 receptor-associated kinase 4	**−2,68**	*eif4ebp1*	Eukaryotic translation initiation factor 4E binding protein 1	**−2,01**

Data are shown as fold change (FC).

Functional categories are indicated in bold.

We further analyzed the transcriptomic effects of exercise training on the fast muscle of adult zebrafish by mining the Ingenuity Knowledge Base for biological functions, pathways and networks. Among the biological functions that showed highly significant (P < 0.00001) changes in fast muscle in response to exercise were muscle development, myogenesis, angiogenesis, cell cycle progression, mitosis, cytoskeleton organization, lipid oxidation, lipid synthesis and organismal growth (Additional file 2: Table S2), with 143, 59, 230, 408, 172, 424, 81, 240 and 201 differentially expressed genes, respectively. The lists of differentialy expressed genes involved in muscle development, myogenesis, angiogenesis and cell proliferation are shown in Additional files 3, 4, 5 and 6: Tables S3-S6. Canonical pathway analysis identified 22 pathways that were significantly (P < 0.05) over-represented in fast muscle of adult exercised zebrafish (Table 4). Regulated canonical signaling pathways associated with skeletal muscle contractile activity included the calcium, integrin, actin cytoskeleton, FGF, wnt/β-catenin and AMPK signaling pathways. Moreover, the IGF-1, insulin receptor, PI3K/AKT and mTOR signaling pathways were also significantly regulated in fast muscle, in accordance with the observed hypertrophy in fast muscle of exercised zebrafish. Interestingly, the canonical TGFβ signaling pathway was also significantly altered by exercise in fast muscle. The metabolic effects of exercise training in the zebrafish fast muscle were exemplified by the significant regulation of the protein ubiquitination pathway, glycolysis and fatty acid β-oxidation. Furthermore, exercise training also caused a significant over-representation of signaling pathways involved in angiogenesis (e.g. ephrin B, VEGF, hypoxia, PDGF, HIF1α, Notch and angiopoietin signaling pathways) in the zebrafish fast muscle (Table 4). The genes that are differentially regulated by exercise training that correspond to each of the over-represented canonical pathways are listed in Additional file 7: Table S7.

**Table 4:** **Significantly over-represented putative canonical pathways in fast muscle of exercised zebrafish**

Ingenuity canonical pathways	p-Value	Ratio
Integrin Signaling	3.28E-16	94/208
Protein Ubiquitination Pathway	3.62E-12	103/268
Wnt/β-catenin Signaling	5.57E-11	75/175
mTOR Signaling	6.06E-06	68/211
TGF-β Signaling	7.22E-06	36/89
Ephrin B Signaling	2.46E-05	32/82
Actin Cytoskeleton Signaling	3.04E-05	72/239
IGF-1 Signaling	6.53E-05	38/105
Glycolysis	1.01E-04	14/41
VEGF Signaling	1.03E-04	36/104
AMPK Signaling	4.67E-04	46/169
Calcium Signaling	6.03E-04	58/213
Insulin Receptor Signaling	1.22E-03	44/142
FGF Signaling	1.53E-03	31/92
Chemokine Signaling	1.72E-03	26/73
PI3K/AKT Signaling	1.75E-03	41/144
Fatty Acid β-oxidation	1.99E-03	14/45
Hypoxia Signaling in the Cardiovascular System	4.35E-03	24/67
PDGF Signaling	4.63E-03	27/85
HIF1α Signaling	9.07E-03	33/108
Notch Signaling	9.60E-03	15/43
Angiopoietin Signaling	3.00E-02	21/74

The associated p-value (Fisher’s exact test P < 0.05) and the ratio of the number of differentially expressed genes in fast muscle of exercised zebrafish over the total number of genes in each particular pathway in the Ingenuity Knowledge Base. Canonical pathway names are from Ingenuity Systems.

**Figure 3:**
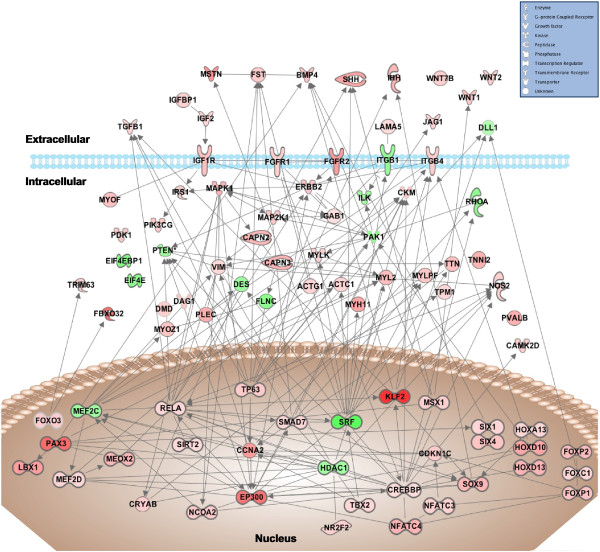
**IPA-based network generated from molecules involved in muscle development and myogenesis that are differentially expressed in fast muscle of exercised adult zebrafish.** The shapes of the genes correlate with the functional classification symbolised in the legend. Arrows represent the direct relationship between molecules. Color intensity correlates to transcription value, calculated as log2ratio (exercised/non-exercised); green represents molecules with repressed transcription (negative log2ratio); red represents molecules with enhanced transcription (positive log2ratio).

**Figure 4:**
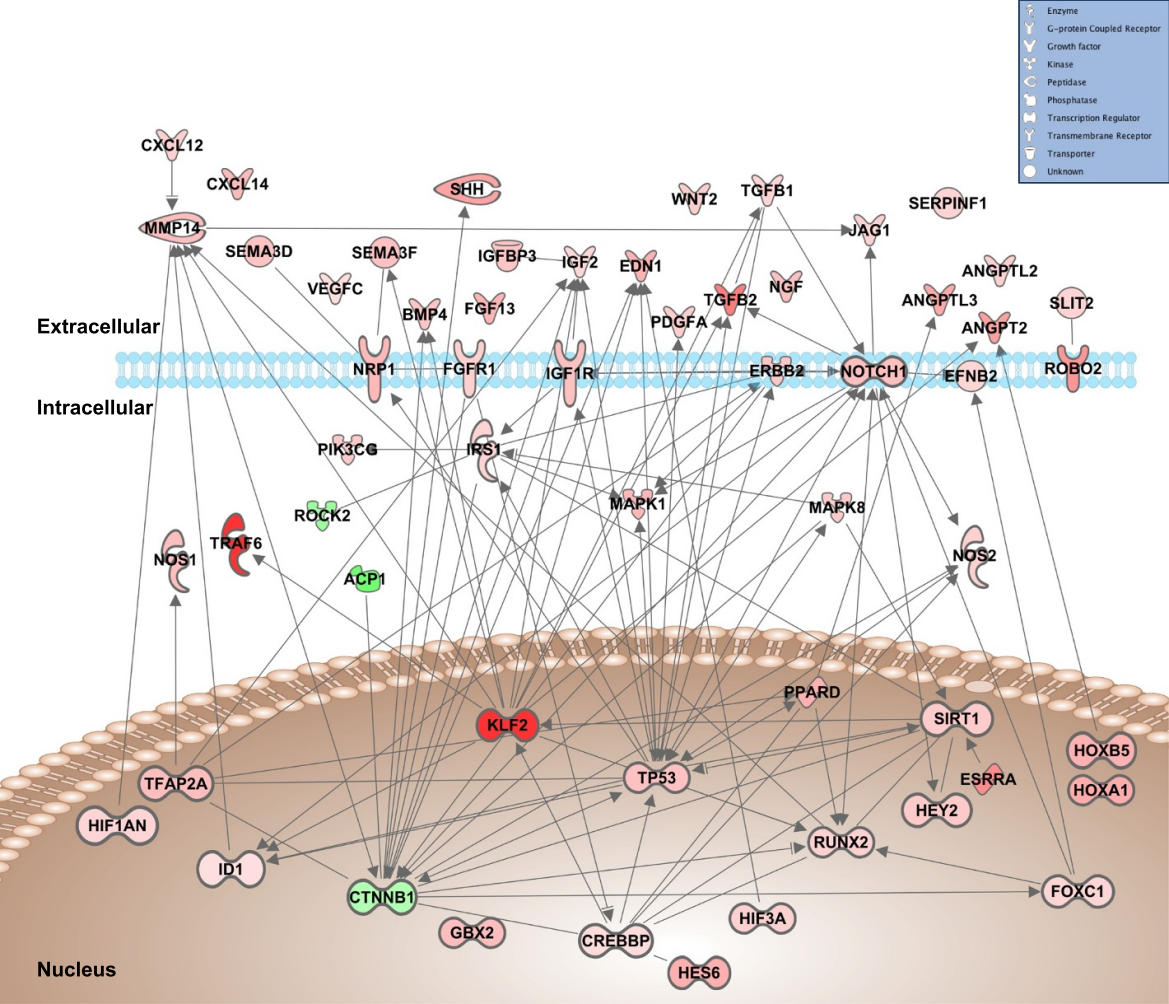
**IPA-based network generated from molecules involved in angiogenesis that are differentially expressed in fast muscle of exercised adult zebrafish.** The shapes of the genes correlate with the functional classification symbolised in the legend. Arrows represent the direct relationship between molecules. Color intensity correlates to transcription value, calculated as log2ratio (exercised/non-exercised); green represents molecules with repressed transcription (negative log2ratio); red represents molecules with enhanced transcription (positive log2ratio).

Analysis of gene networks corresponding to muscle development and angiogenesis by IPA allowed us to establish connectivity maps for these two processes (Figures 3 and 4). The connectivity map of regulated genes involved in muscle development illustrates nodes around transcription factors and nuclear genes such as *ccna2*, *crebbp*, *ep300*, *hdac1*, *kfl2*, *mef2c*, *mef2d*, *pax3*, *rela*, *smad7*, *srf* and *tp63*, that are integrated with key sarcomeric and cytoskeletal elements and key signaling molecules and transducers of extracellular signals involved in the regulation of this process (e.g. *bmp4, dll1, fst, igf2, ihh, jag1, mstn, shh, tgfb1, wnt1, wnt2*) (Figure 3). Regulated genes involved in angiogenesis show a connectivity map with nodes around the nuclear factors *ctnnb1*, *crebbp*, *foxc1*, *klf2*, *runx2*, *tfap2a, tp53* and *sirt1* that are clearly integrated with extracellular signals (e.g. *angpt2, bmp4, edn1, fgf13, igf2, jag1, pdgfa, vegfc*) transducing their effects primarily through the *efnb2*, *erbb2*, *fgf, igf1* and *notch* signaling pathways via molecules such as *irs1*, *mapk1*, *mapk8*, *nos2* and *pik3cg* among others (Figure 4).

The results of microarray analysis were validated by qPCR for 7 differentially expressed genes in fast muscle: 4 down-regulated (*fabp7, tuba1b, psme3, psma5*) and 3 up-regulated (*capns1, fgfrl1, foxa1*) genes. The genes examined showed a similar pattern of change with the two techniques used, except for *capns1* (Additional file 8: Table S8).

## Discussion

### Exercise training induces growth of fast muscle fibers in adult zebrafish

The present study describes the cellular and molecular adaptive mechanisms that are responsible for the plasticity of fast skeletal muscle to exercise-induced contractile activity. Here, we have adopted swimming adult zebrafish as a muscle activity model and have shown, for the first time in adult zebrafish, that exercise training under sustained, aerobic conditions causes hypertrophy of fast muscle fibres. We hypothesize that this may explain, at least in part, the stimulation of muscle growth by swimming in adult zebrafish that we previously reported using the same experimental conditions [17]. Therefore, as in mammals [4, 18] and in other fish species [19], exercise promotes growth in adult zebrafish by increasing muscle mass as a result of increased fibre hypertrophy.

Our gene expression analysis of fast muscle of exercised adult zebrafish shows that the increase in fibre hypertrophy is associated with an important regulation of the fast muscle transcriptome. Here, we show for the first time in zebrafish that exercise-stimulated contractile activity in adult fast muscle induced significant and parallel changes in the expression of canonical pathways important for the regulation of protein turnover, namely the anabolic IGF-1/PI3K/Akt/mTOR signaling pathways that promote protein synthesis and the catabolic ubiquitination and atrophy pathways that are responsible for protein degradation [18]. The increase in the expression of genes involved in protein synthesis and in its regulation (e.g. *igfr1*, *irs1*, *pi3k*, *pdk1*, *pdk2*, *rps6ka1*) and the decrease in the expression of the translation inhibitor *eif4ebp1* (Tables 2 and 3), recently shown to be up-regulated in a zebrafish inactivity model [14], is consistent with the up-regulation of the mRNA expression levels of a large number of genes that code for structural and regulatory contractile elements as well as components of the extracellular matrix in fast muscle of exercised zebrafish. Further support for the activation of this pathway in fast muscle of exercised zebrafish can be found in the down-regulation of the expression of *pten*, a known inhibitor of PI3K/Akt signaling [20]. These observations reinforce the notion that accretion of myofibrillar proteins is an important contributor to muscle growth in fish [21] and strongly suggest that myofibrillogenesis can be stimulated by exercise-induced contractile activity in adult zebrafish. In support of this hypothesis, we recently reported that the increase in protein deposition in the fast muscle of swimming rainbow trout [22] was associated with the transcriptional activation of a large set of genes involved in protein biosynthesis and in muscle contraction and development, including components of the sarcomeric structure of skeletal muscle [23]. Interestingly, in the present study exercise also increased the mRNA expression levels of known regulators of atrophy in skeletal muscle, namely the E3 ubiquitin ligases *trim63* and *fbxo32* [24] and their transcriptional activators *foxo3* [25] and *traf6* [26] (Table 3), consistent with previous reports indicating that TRIM63 and FBXO32 mRNA expression levels increase in hypertrophied muscles in humans subjected to resistance training [27]. These observations suggest that genes involved in the regulation of the degradation of skeletal muscle protein (i.e. atrogenes), in addition to a large set of genes belonging to the ubiquitin proteasome pathway or other proteolytic systems (e.g. calpains), may also participate in the hypertrophic response of the zebrafish fast muscle to exercise-induced contractile activity, possibly to facilitate the maintenance of normal skeletal muscle protein turnover during long-term training [27]. Therefore, our results strongly indicate that exercise-induced hypertrophy of fast muscle fibres in adult zebrafish involves increased protein turnover, shown for the first time in this species by the parallel activation of the IGF-1/PI3K/mTOR signaling and atrophy pathways that, in turn, induce the expression of a number of downstream genes coding for myofibrillar elements, as illustrated by the molecular interactome of the muscle development process (Figure 3).

One of the important and novel findings of our transcriptome analysis of the hypertrophic fast muscle of exercised adult zebrafish is the activation of nearly all TGFβ superfamily signaling pathways known to regulate skeletal muscle mass in mammals. On one hand, we observed an increase in the mRNA levels of follistatin (*fst*), known to promote muscle hypertrophy in mammals by binding myostatin (MSTN) and preventing its interaction with activin receptors resulting in activation of the Akt/mTOR signaling pathway to stimulate protein synthesis [28]. The MSTN signaling pathway, known in mammals and fish to exert a repressive action on muscle hypertrophy [29, 30] through its inhibition of IGF-1/Akt signaling [31], was also up-regulated in fast muscle of exercised zebrafish as evidenced by the increased expression of the extracellular ligand (*mst*), corroborating the results of our previous study [17], receptors (*acvr1b* and *acvr2b*) and signaling molecules (*smad2*). On the other hand, a number of components of the bone morphogenetic protein (BMP) signaling pathway, including extracellular ligands (*bmp1, bmp3, bmp4, bmp8b*), receptors (*mbpr1a, bmpr1b*), gene targets (*id1*) and antagonists such as *noggin* and *smad6*, were also all up-regulated in fast muscle of exercised zebrafish. In mammals, BMPs promote skeletal muscle hypertrophy by stimulating mTOR-dependent anabolism [32, 33]. The results from the present study are significant because they suggest, for the first time, that the BMP signaling pathway may be involved in exercise-induced hypertrophy of skeletal muscle. In mammals, it has been proposed that the regulation of muscle mass depends on the balance between the competing MSTN and BMP signaling pathways [32]. We hypothesize that the exercise-induced increase in muscle mass associated with hypertrophy of fast muscle in adult zebrafish may have resulted, at least in part, from alterations in the normal balance between negative (MSTN) and positive (FST, BMPs) regulators of skeletal muscle mass.

Importantly, our study also provides molecular evidence to suggest that exercise in adult zebrafish may have activated a myogenic program resulting from the activation of satellite cells. Satellite cells, muscle precursor cells with stem cell characteristics [34], are known to contribute importantly to postnatal skeletal muscle growth and muscle regeneration after injury. However, their involvement in hypertrophic muscle growth in adult mammals is currently a subject of debate, particularly in the light of studies showing that hypertrophy does not require the presence of satellite cells [35] or their activation [36, 37]. In contrast, postembryonic muscle growth in zebrafish is accomplished by mosaic hyperplasia (i.e. new myotubes forming on the surface of existing muscle fibres) until fish achieve half of their final body length after which growth is only accomplished by hypertrophy [21]. To date, the exact role of satellite cells (refered to as myogenic precursor cells in fish) in exercise-induced activity in skeletal muscle or whether contractile activity of skeletal muscle fibres can modify the quiescent status of satellite cells and promote their activation in adult muscle are two aspects that are not completely understood. However, there are reports showing that hypertrophy due to resistance training in humans is associated with an increase in the satellite cell pool probably as a result of increased proliferation [38]. Here, we show for the first time in fish that exercise-induced activity in adult zebrafish increased the expression of genes known to participate in the myogenic program, most notably the satellite cell marker *pax3* and its target gene *lbx1*. PAX3 is a key factor in skeletal muscle development thought to be responsible for the enlargement of the satellite cell population in muscle at least in part through its activation of the FGF signaling pathway [4]. PAX3 is important for the activation of the muscle regulatory factors MYOD and, together with the mesenchyme homeobox gene 2 (MEOX2) and SIX proteins (SIX1 and SIX4), of MYF5 [4]. PAX3 was recently shown to be up-regulated specifically in hyperplastic growth zones in the late embryonic myotome in rainbow trout [39], another fish species with hyperplastic growth continuing into adulthood. In the present study, we show that the mRNA expression levels of a number of components of the FGF signaling pathway, including ligands (*fgf13*, *fgf18*, *fgf20*), receptors (*fgfr1*, *fgfr2*, *fgfrl1*) and signaling molecules (*mapk1*, *raf1*, *mapk13*, *crebbp*), as well as *meox2*, *six1* and *six4*, were increased in fast muscle in response to exercise training in adult zebrafish. All these factors interact with *pax3, sox9* and *rela* in a complex molecular network similar to that described in the exercise-trained human skeletal muscle [40, 41]. Interestingly, the canonical Notch and Wnt signaling pathways, known to sequentially control the transition of satellite cells from a proliferative to a differentiative phase [42], were also significantly altered in fast muscle of exercised zebrafish. In accordance with the increased expression of *pax3*, the altered expression of ligands (*dll1*, *jag1*, *jag2*) and receptors (*notch1*, *notch2*) of the Notch signaling pathway, coupled with the significant alteration of the expression of genes involved in mitosis and cell cycle progression (Additional files 6 and 9: Table S6 and Figure S1), suggests that satellite cells may have been activated by exercise. The recent demonstration that satellite cells in adult zebrafish muscle fibres can be activated by mechanical stretch [43] and that *pax3* is expressed in satellite cells isolated from adult zebrafish muscle [44] provide support for the hypothesis that satellite cells may have proliferated in fast muscle of adult zebrafish in response to exercise-induced activity. In addition, exercise caused a significant increase in the expression of components of the Wnt (e.g. *wnt1, wnt2, wnt4, wnt6, wnt7a, wnt7b, wnt8a, wnt10a, wnt10b, wnt11, wnt16; fzd2 to 5, fzd8 to 10; dvl1, dvl2, ccnd1*) and the hedgehog (e.g. *shh, ihh*) signaling pathways, known to play a key role in the induction of myogenesis in vertebrates by promoting differentiation of satellite cells [8, 45]. Interestingly, hyperplastic growth in embryonic trout was also associated with an important up-regulation of growth factors and soluble signaling molecules (including members of the Wnt pathway) [39] but, to our knowledge, this is the first report of exercise regulating the expression of the hedgehog signaling pathway. However, the expression of various paralogs of fast skeletal myosin heavy chain (e.g. myhz1.1, myhz1.2, myhz1.3 and myhz2) that were reported to be markers for hyperplastic growth in zebrafish [15] did not change in fast muscle of exercised adult zebrafish. Therefore, it will be important to investigate in future studies whether exercise can promote proliferation and/or activation of satellite cells in fast muscle of adult zebrafish.

Exercise-induced activity also altered the mRNA expression levels of other important myogenic differentiation factors in the zebrafish fast muscle, most notably Myocyte enhancer factor 2 (*mef2*) and serum response factor (*srf*). MEF2 family members are transcription factors that do not have intrinsic myogenic activity but control the differentiation of skeletal muscle during development through transcriptional cooperation with co-activators such as CREBBP(CBP)/p300, resulting in the potentiation of the function of myogenic regulatory factors (MRFs) and in the regulation of fibre type-specific gene expression programs in mammals [46]. In the adult mammalian muscle, MEF2, in addition to NFAT proteins, is induced by contractile activity in a calcineurin- and CAMKIV-dependent fashion [47] to regulate the metabolic and structural (contractile) phenotype of skeletal muscle cells. Several *mef2* genes are expressed in the zebrafish skeletal muscle [48], with *mef2a* being expressed in fast muscle after differentiation, *mef2c* after myoblast terminal differentiation and *mef2d* in muscle precursor cells [49]. Although Mef2c and Mef2d proteins are not required for muscle fibre terminal differentiation, they are indispensable for myofilament expression and myofibril assembly in zebrafish fast muscle fibres [49]. Recently, *mef2ca* was shown to be induced post-transcriptionally by the TOR pathway to regulate hypertrophic muscle growth in zebrafish [14]. Here, we observed an up-regulation of the mRNA levels of *ep300* and *crebbp*, two nuclear genes that occupy a central position in the transcriptional network in fast muscle of exercised zebrafish (Figure 3), and of *mef2a* and *mef2d*; however, the expression of *mef2ca* was decreased by exercise. In addition, genes involved in calcium signaling initiated by nerve-elicited electrical activity and that regulate MEF2 activity such as *ppp3ca* (calcineurin), its targets *nfatc1*, *nfatc 3* and *nfatc 4*, *camk4* and *hdac4* were all up-regulated by exercise in the zebrafish fast muscle. Another central molecule in the transcriptional network of regulated nuclear genes in the fast muscle of exercised zebrafish is SRF, a transcription factor that regulates myogenic fusion and differentiation and that is also required for overload-induced hypertrophy in the adult mammalian muscle by controlling satellite cell proliferation [50]. The altered expression of *srf* in fast muscle of exercised zebrafish, as well as that of the transcriptional repressor *hdac1*, is consistent with their role as regulators of skeletal myogenesis [50, 51].

### Exercise training promotes vascularization in fast muscle of adult zebrafish

In addition to the increased hypertrophy of fast muscle fibres, exercise increased vascularization of this tissue in adult zebrafish. This is consistent with the well-known increase in capillary number that accompanies fibre hypertrophy in humans and mammalian models [52, 53] and also with previous reports that indicate that swim training increases muscle capillarity in several fish species, including larval zebrafish [54–57]. In mammals, exercise-induced angiogenesis is believed to be induced by the contractile activity of skeletal muscle fibres that, through the combination of growth factor production, hypoxia and shear and mechanical stresses, results in the activation of pro-angiogenic signaling pathways [58]. Importantly, our transcriptomic profiling of the fast muscle of exercised adult zebrafish clearly evidenced the activation of the majority of signaling pathways known in mammals and zebrafish to regulate angiogenesis [59–62], and identified for the first time the molecular programs responsible for the observed increase in vascularization of this tissue by exercise. Specifically, fast skeletal muscle of exercised zebrafish increased the mRNA levels of genes involved in vascular sprouting, including *sema3d*, *sema3f*, *netrin1* and *efnb2,* molecules known to be important for intersegmental vessel formation in zebrafish [62], as well as of *robo2* and *slit2*, an endothelial cell guidance receptor and its ligand, respectively. In addition, exercise also activated at the transcriptional level several canonical signaling pathways known to control the specification of arteries and veins (e.g. Vegf, Notch, Ephrin B2) [63, 64], as supported by the increased mRNA levels of *ssh*, of members of the Vegf signaling pathway including ligands (e.g. *vegfc*), co-receptors (*nrp1*) and downstream signaling molecules (*pik3c2a, pikc3b, pik3cg, plcg1, mapk1*), of *notch1* and of *efnb2* and its receptor *ephb4*. Furthermore, exercise altered the mRNA levels of genes involved in vascular lumen formation in zebrafish such as integrins, *cdc42*, *rac1* and *pax2* [62]. Interestingly, to the best of our knowledge, we provide the first demonstration that exercise increases the mRNA levels in fast muscle of *klf2*, a shear stress-responsive transcription factor that is activated by the onset of blood flow in newly formed vessels and that induces vessel remodelling through alteration of PI3K and MAPK signaling in zebrafish [65]. *klf2* occupies a central position in the angiogenic transcriptional network in fast muscle of exercised adult zebrafish with connections with soluble pro-angiogenic factors (e.g. endothelins, angiopoietins, IGF2, semaphorins), signaling molecules (e.g. *traf6*, *erbb2*) and transcriptional regulators (e.g. *id1*, *ctnnb1*, *crebbp*, *sirt1*) (Figure 4). Remarkably, *klf2*, as well as other components of the angiogenic transcriptional network such as the IGF-1, TGFβ and Notch signaling pathways and the nuclear transcriptional regulator *crebbp*, also participate in the muscle development network (Figure 3). Thus, the molecular response to exercise in skeletal muscle may involve the coordinated activation of angiogenic and muscle development transcriptional programs.

The mechanisms by which angiogenesis is initiated under the normal conditions of adaptive remodelling imposed by exercise are complex and not entirely understood, even in humans. It has been proposed that mechanical and metabolic stimuli responsible for exercise-induced angiogenesis exert their effects by stimulating the production of VEGF, considered to be a central pro-angiogenic factor in the regulation of physiological angiogenesis [52, 66]. In the present study, we report that exercise-induced contractile activity in adult zebrafish caused changes in the expression of the VEGF canonical pathway and of factors that participate in its regulation including members of the hypoxia-inducible factor family (*hif1an*, *hif3a*), nitric oxide synthases (*nos1* and *nos2*), *ppard*, known to increase VEGF production and skeletal muscle angiogenesis [67], and *esrra*, an important mediator of hypoxia-induced PGC-1α transcriptional regulation of VEGF [68]. Therefore, these results suggest that exercise in adult zebrafish may have induced a transcriptional angiogenic program, at least in part, by activating VEGF and its signaling in fast muscle. In support of this hypothesis, swim training in larval zebrafish was recently reported to increase the expression of the HIF and VEGF pathways [69]. To the best of our knowledge, we provide the first evidence that exercise training in zebrafish activates a complex transcriptional program in fast muscle involving multiple signaling pathways (e.g. VEGF, HIF, TGFβ, Ephrin-B, PDGF, angiopoietin) known to participate in the induction and regulation of angiogenesis, resulting in an important increase in vascularization of this tissue.

We hypothesize that, as in mammals [58], the increase in capillarity as a result of exercise training may enhance the exchange of respiratory gasses, substrates and metabolites between the blood and fast muscle. Consequently, by increasing the oxygen exchange capacity and the ensuing oxidative capacity, exercise may induce a more aerobic phenotype in fast muscle in zebrafish, in agreement with previous studies that showed that swim training increased the aerobic capacity of the fast muscle by increasing the expression of respiratory genes in adult zebrafish [70, 71] and in developing zebrafish, as shown by the increased expression of erythropoietin and myoglobin [72]. Support for an increased aerobic phenotype of fast muscle in exercised zebrafish is derived from the observed increased expression of a large set of genes that participate in oxidative metabolism in mitochondria (i.e. TCA cycle and oxidative phosphorylation) and of the oxygen transport protein myoglobin. Although we do not have direct evidence for an effect of exercise on mitochondrial biogenesis, it is interesting to point out that the relationship between capillary and fibre density (C/F ratio), shown here to increase in adult zebrafish in response to exercise as in mammals [58], is related to mitochondrial volume [73] suggesting that swimming-induced exercise could have improved mitochondrial function and number. Surprisingly, the theoretical maximum diffusion distance from the capillaries to the mitochondria increased in fast muscle of exercised zebrafish. Although this finding could initially suggest a reduction in muscle oxidative capacity, it should be only seen as a consequence of fibre hypertrophy. The exercise-induced increase in capillarization of fast fibres relative to their area and perimeter provides further support for the hypothesis of increased mitochondrial oxidative capacity of fast muscle fibres in adult zebrafish subjected to aerobic exercise training.

## Conclusions

In the present study we have shown that exercise-induced contractile activity in adult zebrafish promotes a coordinated adaptive response in fast muscle that leads to increased muscle mass by hypertrophy and increased vascularization by angiogenesis. We hypothesize that these phenotypic adaptations are the result of extensive transcriptional changes induced by exercise. Analysis of the transcriptional networks that are activated in response to exercise in the adult zebrafish fast muscle allowed us to identify signaling pathways and transcriptional regulators that play an important role in the regulation of skeletal muscle mass, myogenesis and angiogenesis by exercise. The present study is the first to describe coordinated molecular programs regulating muscle mass and vascularization induced by exercise in any species other than humans [74] and supports the notion that these programs may regulate “generic” features of exercise adaptation in the vertebrate skeletal muscle. The development of these adaptive responses to exercise in the zebrafish fast muscle, together with an important metabolic remodelling of this tissue, strongly suggest that exercise training may have caused the acquisition of a more aerobic phenotype in fast muscle in zebrafish. It will be interesting to determine in future studies if these changes result in improved aerobic work capacity. In summary, exercise-induced activity resulted in the transcriptional activation of a series of complex networks of extracellular and intracellular signaling molecules and pathways involved in the regulation of muscle mass, myogenesis and angiogenesis in adult zebrafish, some of which had not previously been associated with exercise-induced contractile activity. The results from this study demonstrate the utility of the adult zebrafish as an excellent exercise model for advancing our knowledge on the basic mechanisms underlining the regulation of skeletal muscle mass.

## Methods

### Ethical approval

Experiments complied with the current laws of the Netherlands and were approved by the animal experimental committee (DEC number 09161).

### Experimental fish and conditions

Wild-type zebrafish purchased from a local pet shop were housed in two Blazka-type swim tunnels of 127 liters [17] at 28°C where approximately 500 liters of fresh water were recirculated over a biofilter system. The photoperiod regime was 16L:8D and they were fed twice a day (DuplaRin pellets, Dupla, Gelsdrof, Germany) before and after each daily training session. In total, two separate experiments were performed: Experiment 1 was described previously [17] and Experiment 2 was executed under the exact same conditions. In each of the two experiments, one swim tunnel contained the non-exercised group (Experiment 1: n = 83; Experiment 2: n = 30) and the other tunnel contained the exercised fish (Experiment 1: n = 84; Experiment 2: n = 30).

### Group-wise long term exercise training protocol

In our previous study [17], a swim training protocol was established for adult zebrafish, where the optimal swimming speed (U_opt_) was determined at 0.396 ± 0.019 m s^−1^ or 13.0 ± 0.6 standard body lengths s^−1^. Exercised fish swam at U_opt_ for 6 hours per day during 20 experimental days while non-exercised fish rested at a lower swimming speed of 0.1 m s^−1^. After 20 experimental days, fish were anesthetised with 1 ml clove oil (10% in absolute ethanol) in 1 liter of fresh water and euthanized by decapitation. In Experiment 2, exercised fish showed significantly higher body weight than non-exercised fish (0.34 ± 0.02 g vs. 0.25 ± 0.02 g, P < 0.05), confirming the results of Experiment 1 [17]. Dorsal epaxial fast muscle filets were dissected and either immediately frozen in isopentane cooled to -160°C and stored in liquid nitrogen until sectioned for histochemical analyses (Experiment 2) or stored at -20°C in RNA later (Life Technologies, Barcelona, Spain) for microarray analyses (Experiment 1).

### Muscle histochemical analyses

Fast muscle samples for histochemical analyses were obtained from non-exercised and exercised zebrafish from Experiment 2. After placing the frozen samples in OCT embedding medium at -22°C, serial transverse sections of 16 μm in thickness were obtained in a cryostat (Leica CM3050S, Wetzlar, Germany) and mounted on 2% gelatinised slides. Two histochemical assays were performed on fast muscle serial sections: (1) succinate dehydrogenase (SDH) according to [75] in order to demonstrate the aerobic or anaerobic characteristics of muscle fibres; and (2) endothelial ATPase according to [76] to reveal muscle capillaries.

All morphofunctional measurements of fast muscle cellularity and vascularization were performed on the sections processed for endothelial ATPase activity by using a light microscope (BX61, Olympus, Tokyo, Japan) connected to a digital camera (DP70, Olympus). Image Capturing software (DP Controller v. 1.1.1.65, 2002 Olympus) and Image Managing software (DP Manager v. 1.1.1.71, 2002 Olympus) were used to obtain digital microphotographs and to ensure accurate calibration of all measurements. All the parameters listed below were empirically determined from windows of tissue of approximately 5,5 × · 10^5^ μm^2^ from two different zones or muscle fields in each sample using ImageJ analyzing software (v. 1.47, National Institutes of Health, USA). After testing for the absence of differences between the two muscle fields from each sample, the data obtained from both fields were considered together so that the sample size was large enough. The mean results presented throughout tables and figures were obtained from a sample of n = 8 fish for each condition (non-exercised and exercised).

In order to determine if swimming-induced exercise caused changes in the morphometric and vascularization characteristics of fast muscle fibres, the following parameters were counted or calculated: capillary density (CD; capillary counts per unit cross-sectional area of muscle), fibre density (FD), capillary-to-fibre ratio (C/F = CD/FD; a parameter relatively independent of FCSA and a good indicator of muscle capillarization [73]), the number of capillaries in contact with each fibre (NCF) and the circularity shape factor (SF = 4 · π · FCSA/FPER^2^), which is an estimation of the circular morphology of the fibre (with a value of 1 for a perfect circle). Capillary and fibre counts were calculated and expressed as capillaries and fibres per mm^2^. The following fibre morphometric parameters were measured: fibre cross-sectional area (FCSA) and perimeter (FPER) and the maximal diffusion distance (MDD) between the capillary and the centre of the fibre. The total number of fibres analyzed in each muscle sample ranged from 200 to 250. The indices expressing the relationship between the number of capillaries per fibre and the fibre cross-sectional area (CCA = NCF · 10^3^/FCSA) or fibre perimeter (CCP = NCF · 10^2^/FPER) were also calculated. These indices are considered a measure of the number of capillaries per 1,000 μm^2^ of muscle FCSA and the number of capillaries per 100 μm of muscle FPER. Data for all the parameters are expressed as sample means ± standard error of the mean (SEM).

The histograms of FCSA (Figure 1I-K) express the percentage frequencies of fibres grouped in intervals of 200 μm^2^ and error bars represent the SEM. To obtain the superposed curves in the histograms, a dynamic fitting by nonlinear regression was performed for each group of fish (non-exercised and exercised). The approximation was done by a log-normal (four parameters) equation with a dynamic fit option of 200 for both total number of fits and maximum number of iterations. The *R* values and parameters of the log-normal equations (a, b, x_0_ and y_0_), reported with their SEM, are shown in Additional file 1.

### Microarray analyses

Single color microarray-based gene expression analysis was performed using an Agilent custom oligo microarray 4x44K with eArray design ID 021626 and containing a total of 43.863 probes of 60 oligonucleotides in length. Total RNA from fast skeletal muscle samples of individual adult zebrafish from Experiment 1 (non-exercised, n = 8; exercised, n = 8) was isolated with TRIzol (Invitrogen, Barcelona, Spain). RNA concentrations of the 16 samples used for microarray analyses, as measured with a NanoDrop ND-1000 (Thermo Scientific), ranged from 83 to 260 ng μl^−1^ (134 ± 15 ng μl^−1^), with average absorbance measures (A260/280) of 2.04 ± 0,03, and RNA Integrity Number (RIN) values of 8.85 ± 0.35, as obtained using a 2100 Bioanalyzer system (Agilent Technologies, Santa Clara, CA), that were indicative of clean and intact RNA suitable for microarray analysis. RNA was amplified and labeled with Cy3 dye using single color Low Input Quick Amp Labeling kit (Agilent Technologies) following the manufacturer’s indications using 200 ng of RNA in each reaction. Next, 1.65 μg of labeled cRNA were hybridized to the arrays. Overnight hybridization (17 h, 65°C and 10 rpm rotation) was performed in a Microarray Hybridization Oven (Agilent Technologies). After hybridization, microarrays were washed with Gene Expression Wash Buffers 1 and 2 (Agilent Technologies) and scanned using the High-Resolution C Scanner (Agilent Technologies). Feature Extraction Software 10.7.3 (Agilent Technologies) was used for spot to grid alignment, feature extraction and quantification. Processed data were subsequently imported into GeneSpring GX 11.5 (Agilent Technologies). Significance cut-offs for the ratios of exercised vs non-exercised were set at at P < 0.01 (sample t-test) and >1-fold change for differentially expressed genes (DEGs). For the DEGs, gene IDs were converted to human ENSEMBL gene IDs using g:orth function from G:profiler (http://biit.cs.ut.ee/gprofiler), taking advantage of the more complete gene ontology (GO) annotations of the human genes and improving, in this way, the subsequent analysis of the functional categories. The complete microarray data have been deposited in NCBI´s Gene Expression Omnibus and are accessible through GEO Series accession number GSE58929 (http://www.ncbi.nlm.nih.gov/geo/query/acc.cgi?acc=GSE58929). GO enrichment analysis was performed using Database for Annotation, Visualization and Integrated Discovery (DAVID) software tools (http://david.abcc.ncifcrf.gov), and the resulting categories were considered significant at P < 0.05. Pathway and network analyses were conducted using Ingenuity® Systems Pathway Analysis (IPA) software (Redwood City, CA). To analyze by IPA, annotated spots were mapped to zebrafish and human orthologs using BLASTN against the Ensembl *Danio rerio* gene database (v.Zv9.66) and the *Homo sapiens* transcript database (v.GRCh37.66) with an *e*-value ≤1.00E − 05. Human and zebrafish orthologs were then compared to the Ingenuity® Knowledge Base (http://www.ingenuity.com) and significantly altered pathways and biological functions were determined using the Fisher exact test (P < 0.05).

### Quantitative real-time PCR (qPCR)

Quantitative real time PCR analysis was performed using RNA treated with RQ1 RNase-free DNase (Promega) to remove any contaminating genomic DNA and reverse transcribed using SuperScript III Reverse Transcriptase (Invitrogen), as specified by the manufacturer. Reactions were run in a MyiQ Real-Time PCR Detection System (Bio-Rad, Madrid, Spain) under the following thermal cycling conditions: 2 m at 50°C, 8 min at 95°C, followed by 40 cycles of 15 s denaturation at 95°C and 30 s at corresponding melting temperature, and a final melting curve of 81 cycles from 55°C to 95°C (0.5°C increments every 10 s) to identify the presence of primer dimers and to analyze the specificity of the reaction. The reactions (20 μl) contained 200nM final concentration of each amplification primer, 10μl of SYBR GreenER qPCR SuperMix (Invitrogen) and 5 μl of a 1:25 dilution of cDNA for reference gene and target genes. All PCR reactions were run in triplicate (including the non-template controls) and fluorescence was measured at the end of each extension step. Efficiency of PCR reactions was calculated by analyzing serial dilutions of pooled cDNA samples and was always higher than 99%. The 2^−ΔΔCt^ method [77] was used for real-time PCR analysis and the threshold cycle (Ct) for each gene was normalized to the Ct of RPS15 as reference gene, chosen because of the lack of changes in its expression between exercised and non-exercised zebrafish as assessed by microarray analysis. Primer sequences, amplicon sizes and Ensembl accession numbers of the selected genes are presented in Additional file 10: Table S9.

### Statistical analyses

For capillarization and fibre morphometrical parameters, the normality of the data was tested by the Kolmogorov-Smirnov test (with Lilliefors’ correction) and the comparisons between the two groups of fish (non-exercised and exercised) were analysed by Student’s *t* tests. To test the differences between non-exercised and exercised fish in the frequencies for three intervals of FCSA measured (i.e. fibres with areas below 1.200 μm^2^, between 1.200 and 2.400 μm^2^ and above 2.400 μm^2^; Additional file 1: Table S1), Student’s *t* tests were performed. The normalizing arcsine transformation was applied as a previous step. All statistical analyses were performed using SigmaStat 4.0 (in SigmaPlot 11.0 Software, Systat Software Inc., San Jose, CA, USA).

## Electronic supplementary material

Additional file 1: Table S1: Equation parameters for the log-normal regression of the fiber cross-sectional area histograms in the fast muscle of zebrafish. (PDF 7 KB)

Additional file 2: Table S2: Biological functions that were significantly altered (Fisher’s exact test, p < 0.05) in zebrafish fast muscle in response to swimming. (PDF 14 KB)

Additional file 3: Table S3: List of differentially expressed genes involved in the development of muscle in the zebrafish fast muscle in response to exercise. (PDF 7 KB)

Additional file 4: Table S4: List of differentially expressed genes involved in myogenesis in the zebrafish fast muscle in response to exercise. (PDF 16 KB)

Additional file 5: Table S5: List of differentially expressed genes involved in angiogenesis in the zebrafish fast muscle in response to exercise. (PDF 886 KB)

Additional file 6: Table S6: List of differentially expressed genes involved in cell proliferation in the zebrafish fast muscle in response to exercise. (PDF 23 KB)

Additional file 7: Table S7: Canonical pathways that were significantly altered (Fisher’s exact test, p < 0.05) in zebrafish fast muscle in response to swimming. The number of differentially expressed genes in relation to the total number of genes present in each pathway in the Ingenuity Knowledge Base (No. Genes) and their identity (Pathway molecules) are shown. (PDF 5 KB)

Additional file 8: Table S8: Quantitative real-time PCR (qPCR) validation of microarray results from selected genes. (PDF 360 KB)

Additional file 9: Figure S1: IPA-based network generated from molecules involved in cell proliferation that are differentially expressed in fast muscle of exercised adult zebrafish. The shapes of the genes correlate with the functional classification symbolised in the legend. Arrows represent the direct relationship between molecules. Color intensity correlates to transcription value, calculated as log2ratio (exercised/non-exercised); green represents molecules with repressed transcription (negative log2ratio); red represents molecules with enhanced transcription (positive log2ratio). (PDF 6 KB)

Additional file 10: Table S9: Sequences of primers used in gene expression analyses by qPCR. (PDF 6 KB)
