# Supplementary material for: Swimming-induced exercise promotes hypertrophy and vascularization of fast skeletal muscle fibres and activation of myogenic and angiogenic transcriptional programs in adult zebrafish
Source: BMC Genomics. 2014 Dec 18;15(1):1136. doi: 10.1186/1471-2164-15-1136 (PMC4378002; doi:10.1186/1471-2164-15-1136)
Supplement: Supplementary file 1 — Additional file 1: Table S1: Equation parameters for the log-normal regression of the fiber cross-sectional area histograms in the fast muscle of zebrafish. (PDF 7 KB) [file 12864_2014_6880_MOESM1_ESM.pdf]

Table S2. Biological functions that were significantly altered (Fisher's exact test, $p < 0.05$) in zebrafish fast muscle in response to swimming.

Category	Functions Annotation	p-Value	No. Genes
Skeletal and Muscular System Development and Function	development of muscle	3.44E-09	35/143
Skeletal and Muscular System Development and Function	myogenesis	8.92E-06	24/59
Cardiovascular System Development and Function	angiogenesis	1.35E-07	92/230
Cell Cycle	cell cycle progression	3.57E-17	148/408
Cell Cycle	mitosis	5.53E-09	73/172
Cell Death and Survival	cell survival	1.98E-15	255/488
Cell Signaling	MAPKKK cascade	3.44E-08	23/73
Post-Translational Modification	phosphorylation of protein	2.59E-09	59/254
Cellular Assembly and Organization	microtubule dynamics	8.18E-09	137/356
Cellular Assembly and Organization	organization of cytoskeleton	5.38E-11	148/424
Cellular Development	differentiation of cells	1.71E-13	264/659
Cellular Function and Maintenance	autophagy	7.05E-05	45/96
Gene Expression	transcription of DNA	1.90E-35	247/591
Lipid Metabolism	oxidation of lipid	1.22E-08	43/81
Lipid Metabolism	synthesis of lipid	1.60E-06	99/240
Organismal Development	growth of organism	5.66E-12	64/201
Organismal Development	length of animal	8.80E-09	219/308
Organismal Development	size of body	9.79E-09	220/307
Tissue Morphology	quantity of cells	6.20E-07	289/545
