# Supplementary material for: Swimming-induced exercise promotes hypertrophy and vascularization of fast skeletal muscle fibres and activation of myogenic and angiogenic transcriptional programs in adult zebrafish
Source: BMC Genomics. 2014 Dec 18;15(1):1136. doi: 10.1186/1471-2164-15-1136 (PMC4378002; doi:10.1186/1471-2164-15-1136)
Supplement: Supplementary file 2 — Additional file 2: Table S2: Biological functions that were significantly altered (Fisher’s exact test, p < 0.05) in zebrafish fast muscle in response to swimming. (PDF 14 KB) [file 12864_2014_6880_MOESM2_ESM.pdf]

Table S3. List of differentially expressed genes involved in the development of muscle in the zebrafish fast muscle in response to exercise.

ENSEMBL ID	Gene name	Fold Change
ENSDARG00000031796	ACHE	8.864
ENSDARG00000037840	ACTC1	1.681
ENSDARG00000079111	ACTG1	1.738
ENSDARG00000006901	AEBP1	1.969
ENSDARG00000053493	ALDH1A2	-1.766
ENSDARG00000058868	APC	1.737
ENSG00000165527	ARF6	-1.482
ENSDARG00000019995	BMP4	2.375
ENSDARG00000042552	CACNA1S	2.114
ENSDARG00000014273	CAMK2D	1.974
ENSG00000162909	CAPN2	2.920
ENSDARG00000041864	CAPN3	3.080
ENSDARG00000046004	CAPZB	2.748
ENSDARG00000052004	CAV1	2.317
ENSDARG00000024141	CAV3	-2.626
ENSDARG00000011094	CCNA2	4.132
ENSDARG00000040158	CDC42	-1.971
ENSDARG00000010878	CDKN1C	2.091
ENSG00000064309	CDON	2.387
ENSDARG00000063167	CHKB	-1.767
ENSG00000113163	COL4A3BP	1.843
ENSDARG00000063102	CREBBP	1.410
ENSDARG00000042621	CRYAB	2.050
ENSDARG00000044062	CTBP2	2.318
ENSDARG00000014571	CTNNB1	-1.241
ENSG00000107562	CXCL12	1.898
ENSG00000175084	DES	-1.595
ENSDARG00000009677	DLG1	2.334
ENSDARG00000055115	DTNBP1	-1.760
ENSDARG00000036912	EDN1	3.105
ENSG00000135766	EGLN1	1.895
ENSG00000066044	ELAVL1	-2.341
ENSDARG00000061108	EP300	5.719
ENSDARG00000026294	ERBB2	2.265
ENSDARG00000036041	F2	6.614
ENSDARG00000011027	FGFR1	1.955
ENSDARG00000058115	FGFR2	4.047
ENSG00000127418	FGFRL1	4.323
ENSDARG00000022684	FKBP1A	-1.506
ENSG00000128591	FLNC	-1.883
ENSDARG00000040623	FOSL2	2.001
ENSG00000054598	FOXC1	1.683
ENSDARG00000014181	FOXP1	2.497
ENSG00000128573	FOXP2	2.758
ENSG00000134363	FST	2.166
ENSDARG00000037018	GAB1	1.317
ENSDARG00000015427	HDAC1	-1.474
ENSDARG00000070538	HEY1	1.782
ENSG00000106031	HOXA13	1.633
ENSDARG00000057859	HOXD10	3.644
ENSG00000128714	HOXD13	3.042
ENSDARG00000020241	ICMT	-1.846
ENSDARG00000027423	IGF1R	2.532
ENSDARG00000018643	IGF2	1.613
ENSDARG00000058733	IHH	2.909
ENSDARG00000056964	ILK	-1.538
ENSDARG00000035350	INS	1.908
ENSDARG00000053255	ITGB1	-1.702
ENSG00000101384	JAG1	1.804
ENSDARG00000058603	JPH1	1.550
ENSG00000127528	KLF2	8.516
ENSDARG00000023082	KRT17	8.402
ENSDARG00000056043	LAMA1	2.179
ENSDARG00000058543	LAMA5	1.393
ENSG00000138136	LBX1	4.027
ENSDARG00000015824	LEMD3	1.595
ENSDARG00000055903	LUC7L	1.940
ENSDARG00000007825	MAP2K1	2.304
ENSDARG00000005416	MAP3K5	1.428
ENSDARG00000027552	MAPK1	2.995
ENSDARG00000000857	MAPK14	-2.781
ENSDARG00000023110	MAPK7	3.215
ENSG00000175130	MARCKSL1	-1.561
ENSG00000081189	MEF2C	-1.593
ENSDARG00000040237	MEF2D	1.711
ENSDARG00000040911	MEOX2	2.967
ENSDARG00000008388	MMP14	2.687
ENSDARG00000070546	MSGN1	2.129
ENSDARG00000069133	MSTN	4.409
ENSDARG00000007641	MSX1	2.004
ENSDARG00000009782	MYH11	3.626
ENSDARG00000053424	MYL2	2.639
ENSG00000065534	MYLK	1.871
ENSG00000180209	MYLPF	2.255
ENSDARG00000017128	MYOF	2.490
ENSDARG00000071445	MYOZ1	2.405
ENSDARG00000079475	NCKIPSD	3.167
ENSDARG00000017929	NCOA2	2.125
ENSDARG00000008937	NEO1	1.620
ENSDARG00000010047	NEU3	1.405
ENSDARG00000076297	NFATC3	1.919
ENSG00000100968	NFATC4	3.080
ENSDARG00000020053	NKD1	1.902
ENSDARG00000068910	NOS1	2.649
ENSDARG00000026925	NOS2	1.851
ENSG00000185551	NR2F2	1.947
ENSDARG00000045373	PABPN1	-1.966
ENSG00000182752	PAPPA	3.094
ENSDARG00000010192	PAX3	6.205
ENSDARG00000055505	PDGFA	2.024
ENSDARG00000062590	PLEC	3.313
ENSDARG00000056623	PTEN	-1.535
ENSDARG00000020334	PTPN11	1.135
ENSDARG00000034705	PVALB	3.239
ENSDARG00000074849	RAC1	-1.574
ENSDARG00000077505	RBP4	1.899
ENSG00000173039	RELA	1.678
ENSDARG00000070047	RGS4	1.545
ENSDARG00000094673	RHOA	-1.825
ENSDARG00000004301	RHOG	-2.467
ENSDARG00000006553	RRAS	-1.524
ENSDARG00000005775	SCN8A	2.124
ENSDARG00000068567	SHH	3.784
ENSDARG00000011488	SIRT2	1.477
ENSDARG00000026473	SIX1	1.749
ENSDARG00000004695	SIX4	2.393
ENSDARG00000034268	SLIT3	2.525
ENSDARG00000016858	SMAD7	1.631
ENSG00000072195	SPEG	2.225
ENSDARG00000053918	SRF	-2.680
ENSDARG00000060723	STIM1	-1.670
ENSDARG00000004906	STIP1	-2.666
ENSDARG00000051874	STRA6	1.652
ENSDARG00000006120	TBX2	1.661
ENSG00000089225	TBX5	1.742
ENSDARG00000041502	TGFB1	1.706
ENSDARG00000029995	TNNI2	3.119
ENSDARG00000044356	TP63	2.158
ENSDARG00000087402	Tpm1	1.281
ENSG00000140416	TPM1	1.706
ENSG00000038382	TRIO	2.563
ENSG00000155657	TTN	2.577
ENSDARG00000010008	VIM	1.614
ENSDARG00000014113	WASL	1.372
ENSDARG00000055554	WNT1	2.278
ENSDARG00000040925	WNT10B	2.230
ENSDARG00000005050	WNT2	1.967
ENSDARG00000071107	WNT7B	1.952
ENSDARG00000031420	WT1	2.456
ENSDARG00000002271	ZFAND5	1.498
ENSG00000140836	ZFHX3	2.040
ENSDARG00000041572	ZFPM1	2.902
ENSDARG00000040123	ZFPM2	2.548
