# Supplementary material for: Swimming-induced exercise promotes hypertrophy and vascularization of fast skeletal muscle fibres and activation of myogenic and angiogenic transcriptional programs in adult zebrafish
Source: BMC Genomics. 2014 Dec 18;15(1):1136. doi: 10.1186/1471-2164-15-1136 (PMC4378002; doi:10.1186/1471-2164-15-1136)
Supplement: Supplementary file 3 — Additional file 3: Table S3: List of differentially expressed genes involved in the development of muscle in the zebrafish fast muscle in response to exercise. (PDF 7 KB) [file 12864_2014_6880_MOESM3_ESM.pdf]

Table S4. List of differentially expressed genes involved in myogenesis in the zebrafish fast muscle in response to exercise.

ENSEMBL ID	Gene name	Fold Change
ENSDARG00000079111	ACTG1	1.738
ENSG00000165527	ARF6	-1.482
ENSDARG00000019995	BMP4	2.375
ENSDARG00000042552	CACNA1S	2.114
ENSDARG00000014273	CAMK2D	1.974
ENSG00000162909	CAPN2	2.920
ENSDARG00000041864	CAPN3	3.080
ENSDARG00000024141	CAV3	-2.626
ENSDARG00000040158	CDC42	-1.971
ENSG00000064309	CDON	2.387
ENSG00000113163	COL4A3BP	1.843
ENSDARG00000014571	CTNNB1	-1.241
ENSDARG00000036912	EDN1	3.105
ENSG00000066044	ELAVL1	-2.341
ENSDARG00000061108	EP300	5.719
ENSDARG00000036041	F2	6.614
ENSDARG00000011027	FGFR1	1.955
ENSG00000128591	FLNC	-1.883
ENSDARG00000040623	FOSL2	2.001
ENSDARG00000015427	HDAC1	-1.474
ENSDARG00000070538	HEY1	1.782
ENSDARG00000018643	IGF2	1.613
ENSDARG00000035350	INS	1.908
ENSDARG00000053255	ITGB1	-1.702
ENSDARG00000056043	LAMA1	2.179
ENSDARG00000015824	LEMD3	1.595
ENSDARG00000007825	MAP2K1	2.304
ENSDARG00000027552	MAPK1	2.995
ENSG00000175130	MARCKSL1	-1.561
ENSG00000081189	MEF2C	-1.593
ENSDARG00000040237	MEF2D	1.711
ENSDARG00000040911	MEOX2	2.967
ENSDARG00000008388	MMP14	2.687
ENSDARG00000069133	MSTN	4.409
ENSDARG00000053424	MYL2	2.639
ENSG00000065534	MYLK	1.871
ENSDARG00000017128	MYOF	2.490
ENSDARG00000071445	MYOZ1	2.405
ENSDARG00000079475	NCKIPSD	3.167
ENSDARG00000008937	NEO1	1.620
ENSDARG00000010047	NEU3	1.405
ENSDARG00000068910	NOS1	2.649
ENSDARG00000026925	NOS2	1.851
ENSDARG00000010192	PAX3	6.205
ENSDARG00000055505	PDGFA	2.024
ENSDARG00000020334	PTPN11	1.135
ENSDARG00000074849	RAC1	-1.574
ENSG00000173039	RELA	1.678
ENSDARG00000094673	RHOA	-1.825
ENSDARG00000004301	RHOG	-2.467
ENSDARG00000026473	SIX1	1.749
ENSDARG00000053918	SRF	-2.680
ENSG00000089225	TBX5	1.742
ENSDARG00000041502	TGFB1	1.706
ENSG00000140416	TPM1	1.706
ENSG00000038382	TRIO	2.563
ENSG00000155657	TTN	2.577
ENSDARG00000014113	WASL	1.372
ENSDARG00000055554	WNT1	2.278
