# Supplementary material for: Swimming-induced exercise promotes hypertrophy and vascularization of fast skeletal muscle fibres and activation of myogenic and angiogenic transcriptional programs in adult zebrafish
Source: BMC Genomics. 2014 Dec 18;15(1):1136. doi: 10.1186/1471-2164-15-1136 (PMC4378002; doi:10.1186/1471-2164-15-1136)
Supplement: Supplementary file 4 — Additional file 4: Table S4: List of differentially expressed genes involved in myogenesis in the zebrafish fast muscle in response to exercise. (PDF 16 KB) [file 12864_2014_6880_MOESM4_ESM.pdf]

Table S5. List of differentially expressed genes involved in angiogenesis in the zebrafish fast muscle in response to exercise.

ENSEMBL ID	Gene name	Fold Change	ENSEMBL ID	Gene name	Fold Change
ENSDARG00000016918	ACE2	1.605	ENSDARG00000056633	FGF13	3.014
ENSDARG00000076486	ACP1	-2.308	ENSDARG00000011027	FGFR1	1.955
ENSDARG00000044422	ACVR2B	1.737	ENSDARG00000058115	FGFR2	4.047
ENSDARG00000019819	ADRA2B	2.092	ENSG00000054598	FOXC1	1.683
ENSDARG00000060109	AGGF1	-2.098	ENSDARG00000042904	FOXO3	2.078
ENSDARG00000053493	ALDH1A2	-1.766	ENSDARG00000062909	FURIN	-2.539
ENSDARG00000078335	AMOT	2.34	ENSDARG00000077111	FZD4	2.116
ENSDARG00000053906	ANGPT2	3.902	ENSDARG00000025420	FZD5	1.962
ENSDARG00000024030	ANGPTL2	1.902	ENSDARG00000037018	GAB1	1.317
ENSDARG00000044365	ANGPTL3	3.467	ENSDARG00000069991	GADD45A	-2.781
ENSDARG00000058504	AP1S2	-1.53	ENSG00000183087	GAS6	3.02
ENSDARG00000058868	APC	1.737	ENSDARG00000013477	GATA1	2.999
ENSDARG00000012076	APOA1	2.408	ENSDARG00000002933	GBX2	2.693
ENSDARG00000042780	APOB	2.66	ENSDARG00000018953	GCLM	-1.526
ENSDARG00000040295	APOE	-1.906	ENSDARG00000052131	GLI3	4.671
ENSDARG00000043795	ARHGDIA	-1.49	ENSDARG00000058267	GLMN	-1.526
ENSDARG00000035732	ARNTL	-1.973	ENSG00000120063	GNA13	-2.361
ENSDARG00000019995	BMP4	2.375	ENSDARG00000011459	GSN	-1.584
ENSDARG00000045097	BMPR1A	2.584	ENSDARG00000018441	HEG1	2.003
ENSDARG00000017661	BRAF	5.846	ENSDARG00000036482	HEXIM1	2.821
ENSDARG00000051955	BRMS1	-1.984	ENSDARG00000070538	HEY1	1.782
ENSDARG00000055585	C1GALT1	1.683	ENSDARG00000013441	HEY2	2.196
ENSG00000179218	CALR	-2.407	ENSDARG00000041169	HIF3A	1.919
ENSDARG00000013804	CAPNS1	-1.288	ENSDARG00000029722	HMGB2	1.81
ENSDARG00000052004	CAV1	2.317	ENSG00000105991	HOXA1	3.435
ENSDARG00000040158	CDC42	-1.971	ENSG00000106031	HOXA13	1.633
ENSDARG00000018693	CDH2	-1.418	ENSDARG00000029263	HOXB3	1.911
ENSDARG00000075211	CHD7	2.916	ENSDARG00000013057	HOXB5	3.213
ENSDARG00000008829	CHGA	1.964	ENSDARG00000092809	HOXC9	3.07
ENSDARG00000077769	CITED1	1.913	ENSDARG00000057859	HOXD10	3.644
ENSDARG00000022995	CLIC4	-1.5	ENSDARG00000054754	HS6ST1	2.125
ENSG00000182871	COL18A1	2.595	ENSDARG00000014907	HTRA1	2.36
ENSDARG00000012405	COL1A1	2.694	ENSDARG00000040764	ID1	1.311
ENSDARG00000055009	COL4A1	1.965	ENSDARG00000054823	ID3	-2.39
ENSG00000171812	COL8A2	12.056	ENSDARG00000027423	IGF1R	2.532
ENSDARG00000031203	COMMD1	1.579	ENSDARG00000018643	IGF2	1.613
ENSDARG00000063102	CREBBP	1.41	ENSDARG00000014859	IGFBP3	1.661
ENSDARG00000028699	CRKL	-1.89	ENSDARG00000017389	IGFBP7	2.363
ENSDARG00000042621	CRYAB	2.05	ENSDARG00000058733	IHH	2.909
ENSDARG00000044062	CTBP2	2.318	ENSDARG00000039436	IL13RA2	2.54
ENSDARG00000074301	CTH	2.717	ENSDARG00000090344	IL20	2.437
ENSDARG00000014571	CTNNB1	-1.241	ENSDARG00000030716	ING4	1.62
ENSDARG00000055120	CTSB	2.16	ENSDARG00000035350	INS	1.908
ENSG00000107562	CXCL12	1.898	ENSDARG00000054087	IRS1	1.775
ENSDARG00000056627	CXCL14	2.833	ENSDARG00000053255	ITGB1	-1.702
ENSDARG00000018283	CYBA	-2.958	ENSDARG00000016939	ITGB2	2.257
ENSDARG00000042014	CYP11B2	2.291	ENSG00000101384	JAG1	1.804
ENSDARG00000023062	CYR61	1.753	ENSDARG00000074378	JUNB	-2.259
ENSG00000153071	DAB2	3.596	ENSG00000127528	KLF2	8.516
ENSDARG00000069591	DDAH1	2.009	ENSDARG00000007149	L1CAM	4.503
ENSDARG00000035564	DGCR8	-1.788	ENSDARG00000056043	LAMA1	2.179
ENSDARG00000060626	DGKA	1.845	ENSDARG00000015824	LEMD3	1.595
ENSG00000107984	DKK1	4.238	ENSDARG00000068365	LMX1B	1.691
ENSDARG00000009677	DLG1	2.334	ENSDARG00000042561	LPAR2	5.502
ENSDARG00000020219	DLL1	-1.248	ENSDARG00000033604	LRPAP1	-2.113
ENSDARG00000055563	DROSHA	1.668	ENSDARG00000007825	MAP2K1	2.304
ENSDARG00000010432	EAF2	-2.073	ENSDARG00000027552	MAPK1	2.995
ENSDARG00000036912	EDN1	3.105	ENSDARG00000000857	MAPK14	-2.781
ENSG00000125266	EFNB2	1.634	ENSDARG00000023110	MAPK7	3.215
ENSDARG00000013847	EGFR	-2.134	ENSG00000107643	MAPK8	2.124
ENSG00000135766	EGLN1	1.895	ENSDARG00000031952	MB	2.949
ENSDARG00000057671	EPAS1	2.361	ENSDARG00000057513	MDM4	1.658
ENSDARG00000027112	EPHB4	1.965	ENSG00000081189	MEF2C	-1.593
ENSDARG00000021859	ERAP1	1.628	ENSDARG00000089456	MEN1	1.366
ENSDARG00000026294	ERBB2	2.265	ENSDARG00000040911	MEOX2	2.967
ENSDARG00000004111	ESR1	1.94	ENSG00000140545	MFGE8	3.102
ENSG00000134954	ETS1	-1.955	ENSDARG00000012407	MGAT1	1.633
ENSDARG00000036041	F2	6.614	ENSDARG00000071506	MGAT5	2.645
ENSDARG00000021013	F3	6.286	ENSDARG00000008388	MMP14	2.687
			ENSDARG00000007241	MYC	-2.267

Supplementary Table 5 (Cont.). List of differentially expressed genes involved in angiogenesis in the zebrafish fast muscle in response to exercise.

ENSEMBL ID	Gene name	Fold Change
ENSDARG00000017128	MYOF	2.49
ENSDARG00000079475	NCKIPSD	3.167
ENSDARG00000076297	NFATC3	1.919
ENSG00000100968	NFATC4	3.08
ENSG00000134259	NGF	2.548
ENSDARG00000019835	NKX2-1	2.883
ENSG00000119919	NKX2-3	1.924
ENSG00000183691	NOG	4.897
ENSDARG00000068910	NOS1	2.649
ENSDARG00000026925	NOS2	1.851
ENSG00000148400	NOTCH1	2.6
ENSDARG00000043130	NOTCH2	-1.892
ENSDARG00000042850	NPHS2	2.835
ENSDARG00000036222	NPY	2.877
ENSG00000185551	NR2F2	1.947
ENSDARG00000000796	NR4A1	2.189
ENSDARG00000071865	NRP1	2.888
ENSG00000065320	NTN1	1.647
ENSDARG00000007377	ODC1	-4.792
ENSDARG00000057702	OGT	-1.512
ENSDARG00000042845	OXT	4.038
ENSG00000149269	PAK1	-1.865
ENSDARG00000017703	PAQR3	-1.523
ENSDARG00000089610	PBX1	1.458
ENSG00000114209	PDCD10	-1.322
ENSDARG00000055505	PDGFA	2.024
ENSDARG00000032780	PIGF	-2.367
ENSDARG00000060841	PIK3C2A	2.966
ENSDARG00000086927	PIK3C2B	3.06
ENSG00000105851	PIK3CG	2.211
ENSDARG00000059120	PIM1	1.955
ENSDARG00000034080	PLCD1	1.659
ENSDARG00000089038	PLEKHG5	-1.463
ENSDARG00000060715	PLOD3	-1.456
ENSDARG00000019328	PLXNA4	2.068
ENSDARG00000055092	POR	2.573
ENSDARG00000059933	PPAP2B	4.028
ENSDARG00000091024	PPARA	2.766
ENSDARG00000042247	PPIA	-1.63
ENSG00000221823	PPP3R1	-1.596
ENSDARG00000070651	PRKCD	-1.378
ENSDARG00000003008	PRKCE	4.183
ENSDARG00000075083	PRKDC	-1.51
ENSDARG00000020656	PRKG1	2.535
ENSDARG00000013968	PSAP	-1.344
ENSDARG00000004870	PSEN1	-1.324
ENSDARG00000056623	PTEN	-1.535
ENSDARG00000007923	PTPN1	-1.554
ENSDARG00000079515	PTX3	2.529
ENSDARG00000074849	RAC1	-1.574
ENSDARG00000021550	RAD23B	7.21
ENSG00000132155	RAF1	2.311
ENSG00000116473	RAP1A	-1.972
ENSDARG00000008867	RAP1B	-1.465
ENSG00000109756	RAPGEF2	1.885
ENSDARG00000034893	RARA	1.927
ENSDARG00000034117	RARB	1.832
ENSG00000185989	RASA3	-1.77
ENSDARG00000045636	RBL2	1.41
ENSDARG00000073847	RBM15	1.961
ENSG00000173039	RELA	1.678
ENSDARG00000020544	REM1	1.712

ENSEMBL ID	Gene name	Fold Change
ENSDARG00000035810	RGCC	3.04
ENSDARG00000070047	RGS4	1.545
ENSDARG00000002644	RGS5	1.93
ENSDARG00000094673	RHOA	-1.825
ENSDARG00000015429	RHOB	-1.396
ENSDARG00000004301	RHOG	-2.467
ENSDARG00000020250	RHOJ	1.678
ENSG00000124813	RUNX2	1.899
ENSDARG00000009978	S100A4	-1.626
ENSDARG00000042690	S1PR1	2.034
ENSDARG00000008237	SARS	-2.601
ENSDARG00000042697	SCARB1	6.121
ENSDARG00000038574	SCG2	8.03
ENSDARG00000042138	SELP	2.33
ENSDARG00000011163	SEMA3F	2.569
ENSDARG00000042684	SERPINC1	3.484
ENSDARG00000069048	SERPINF1	1.792
ENSG00000107338	SHB	2.487
ENSDARG00000068567	SHH	3.784
ENSDARG00000068064	SIRT1	2.153
ENSDARG00000004246	SLIT2	1.791
ENSDARG00000034268	SLIT3	2.525
ENSDARG00000016858	SMAD7	1.631
ENSDARG00000077226	SMARCA4	3.979
ENSDARG00000002952	SMO	2.002
ENSG00000112562	SMOC2	1.955
ENSG00000164736	SOX17	2.288
ENSDARG00000041449	SPRED1	1.938
ENSDARG00000068732	SPRY4	3.63
ENSDARG00000053918	SRF	-2.68
ENSDARG00000076484	STAB1	1.574
ENSDARG00000060723	STIM1	-1.67
ENSDARG00000011312	STK3	-1.586
ENSDARG00000051874	STRA6	1.652
ENSDARG00000008186	SYK	-2.766
ENSG00000055208	TAB2	2.584
ENSDARG00000019930	TAL1	2.645
ENSG00000139372	TDG	1.764
ENSDARG00000059279	TFAP2A	2.688
ENSDARG00000034718	TFPI	2.035
ENSDARG00000041502	TGFB1	1.706
ENSG00000092969	TGFB2	5.352
ENSDARG00000073810	THBS2	1.572
ENSDARG00000000151	THRA	2.052
ENSG00000041982	TNC	2.531
ENSDARG00000018569	TNFRSF1A	-1.346
ENSDARG00000035559	TP53	2.532
ENSDARG00000028058	TRAF6	10.775
ENSDARG00000069640	VEGFC	1.361
ENSDARG00000008247	VEZF1	2.618
ENSDARG00000010008	VIM	1.614
ENSG00000140105	WARS	-2.656
ENSDARG00000011801	WARS2	1.783
ENSDARG00000014796	WNT11	2.167
ENSDARG00000005050	WNT2	1.967
ENSDARG00000031420	WT1	2.456
ENSDARG00000016143	XIAP	1.378
ENSDARG00000035913	YARS	-1.889
ENSDARG00000071658	YWHAG	-2.565
ENSDARG00000042539	YWHAZ	3.237
ENSDARG00000016154	ZFP36L1	4.411
ENSDARG00000040123	ZFPM2	2.548
