# Supplementary material for: Swimming-induced exercise promotes hypertrophy and vascularization of fast skeletal muscle fibres and activation of myogenic and angiogenic transcriptional programs in adult zebrafish
Source: BMC Genomics. 2014 Dec 18;15(1):1136. doi: 10.1186/1471-2164-15-1136 (PMC4378002; doi:10.1186/1471-2164-15-1136)
Supplement: Supplementary file 5 — Additional file 5: Table S5: List of differentially expressed genes involved in angiogenesis in the zebrafish fast muscle in response to exercise. (PDF 886 KB) [file 12864_2014_6880_MOESM5_ESM.pdf]

Table S6. List of differentially expressed genes involved in cell proliferation in the zebrafish fast muscle in response to exercise.

ENSEMBL ID	Gene name	Fold Change	ENSEMBL ID	Gene name	Fold Change
ENSDARG00000014031	ABCC2	2.072	ENSDARG00000007825	MAP2K1	2.304
ENSDARG00000016918	ACE2	1.605	ENSDARG00000027552	MAPK1	2.995
ENSDARG00000053493	ALDH1A2	-1.766	ENSDARG00000000857	MAPK14	-2.781
ENSDARG00000054755	ALOX5AP	2.822	ENSDARG00000070573	MAPK3	1.405
ENSDARG00000053906	ANGPT2	3.902	ENSDARG00000079504	MFN2	2.424
ENSDARG00000040295	APOE	-1.906	ENSDARG00000071336	MIF	-1.495
ENSDARG00000019995	BMP4	2.375	ENSDARG00000008388	MMP14	2.687
ENSDARG00000053617	CAMK2A	1.977	ENSDARG00000002077	MNAT1	-1.469
ENSDARG00000014273	CAMK2D	1.974	ENSDARG00000069133	MSTN	4.409
ENSG00000162909	CAPN2	2.920	ENSDARG00000053666	MYB	2.888
ENSDARG00000052004	CAV1	2.317	ENSDARG00000007241	MYC	-2.267
ENSG00000115355	CCDC88A	4.979	ENSDARG00000006837	MYCN	2.067
ENSDARG00000011094	CCNA2	4.132	ENSDARG00000010169	MYD88	2.564
ENSDARG00000035750	CCND1	1.863	ENSDARG00000012378	NAA35	1.577
ENSDARG00000017602	CCNG2	2.293	ENSDARG00000030598	NAMPT	-2.451
ENSDARG00000068214	CCNI	1.385	ENSDARG00000020872	NFAT5	1.587
ENSDARG00000034146	CCNK	-1.689	ENSDARG00000036168	NFATC1	2.762
ENSG00000008128	CDK11	-1.567	ENSDARG00000076297	NFATC3	1.919
ENSDARG00000063726	CDK12	2.259	ENSG00000183691	NOG	4.897
ENSDARG00000032072	CDK16	2.979	ENSDARG00000026925	NOS2	1.851
ENSDARG00000051916	CDK7	-1.458	ENSG00000148400	NOTCH1	2.600
ENSDARG00000016496	CDK8	-2.007	ENSDARG00000010250	NQO1	1.935
ENSDARG00000044811	CDK9	1.839	ENSDARG00000000796	NR4A1	2.189
ENSDARG00000010878	CDKN1C	2.091	ENSG00000153234	NR4A2	1.781
ENSDARG00000087303	CEBPD	1.903	ENSDARG00000055854	NR4A3	1.957
ENSDARG00000025679	COMT	2.255	ENSDARG00000007377	ODC1	-4.792
ENSDARG00000053586	CREB1	-1.313	ENSDARG00000042845	OXT	4.038
ENSDARG00000014571	CTNNB1	-1.241	ENSG00000182752	PAPPA	3.094
ENSDARG00000038954	CTNNBIP1	-1.933	ENSDARG00000052025	POMGNT1	-1.787
ENSDARG00000035564	DGCR8	-1.788	ENSDARG00000061219	POSTN	1.888
ENSDARG00000070463	E2F3	1.823	ENSDARG00000091024	PPARA	2.766
ENSDARG00000036912	EDN1	3.105	ENSDARG00000044525	PPARD	3.189
ENSDARG00000013847	EGFR	-2.134	ENSDARG00000042247	PPIA	-1.630
ENSG00000066044	ELAVL1	-2.341	ENSDARG00000020656	PRKG1	2.535
ENSDARG00000004111	ESR1	1.940	ENSDARG00000056623	PTEN	-1.535
ENSDARG00000036041	F2	6.614	ENSDARG00000069439	PTGDS	3.184
ENSDARG00000021013	F3	6.286	ENSDARG00000020334	PTPN11	1.135
ENSDARG00000058115	FGFR2	4.047	ENSDARG00000029663	RAB1A	-1.808
ENSDARG00000014181	FOXP1	2.497	ENSDARG00000078653	RALBP1	-1.691
ENSG00000134363	FST	2.166	ENSDARG00000034893	RARA	1.927
ENSDARG00000077111	FZD4	2.116	ENSDARG00000045636	RBL2	1.410
ENSDARG00000053326	GNA11	5.086	ENSDARG00000077505	RBP4	1.899
ENSG00000156052	GNAQ	3.248	ENSDARG00000070047	RGS4	1.545
ENSG00000198873	GRK5	2.978	ENSDARG00000094673	RHOA	-1.825
ENSG00000197921	HES5	2.113	ENSG00000134318	ROCK2	-1.610
ENSDARG00000070538	HEY1	1.782	ENSDARG00000002006	RXRB	1.792
ENSDARG00000013441	HEY2	2.196	ENSDARG00000042690	S1PR1	2.034
ENSDARG00000001975	HSD11B2	1.714	ENSDARG00000008188	SF1	-1.395
ENSDARG00000056160	HSPD1	-3.237	ENSDARG00000068567	SHH	3.784
ENSDARG00000054823	ID3	-2.390	ENSDARG00000077226	SMARCA4	3.979
ENSDARG00000027423	IGF1R	2.532	ENSDARG00000088347	SP1	1.763
ENSDARG00000018643	IGF2	1.613	ENSDARG00000019353	SPARC	2.589
ENSDARG00000038666	IGFBP1	1.892	ENSDARG00000055588	STAT5B	1.968
ENSDARG00000014859	IGFBP3	1.661	ENSG00000089225	TBX5	1.742
ENSDARG00000033727	IL12B	3.681	ENSDARG00000034718	TFPI	2.035
ENSDARG00000056964	ILK	-1.538	ENSDARG00000041502	TGFB1	1.706
ENSDARG00000010657	IRAK4	-2.680	ENSG00000137462	TLR2	2.268
ENSDARG00000032768	IRF1	2.232	ENSG00000041982	TNC	2.531
ENSDARG00000053255	ITGB1	-1.702	ENSDARG00000018569	TNFRSF1A	-1.346
ENSDARG00000074378	JUNB	-2.259	ENSDARG00000035559	TP53	2.532
ENSG00000160145	KALRN	3.395	ENSDARG00000028058	TRAF6	10.775
ENSDARG00000056043	LAMA1	2.179	ENSDARG00000031435	TXN	-1.896
ENSDARG00000042561	LPAR2	5.502	ENSDARG00000020008	VCP	-1.820
ENSDARG00000029146	LRP1	-2.220	ENSDARG00000042642	WTAP	-1.220
