# Supplementary material for: Swimming-induced exercise promotes hypertrophy and vascularization of fast skeletal muscle fibres and activation of myogenic and angiogenic transcriptional programs in adult zebrafish
Source: BMC Genomics. 2014 Dec 18;15(1):1136. doi: 10.1186/1471-2164-15-1136 (PMC4378002; doi:10.1186/1471-2164-15-1136)
Supplement: Supplementary file 6 — Additional file 6: Table S6: List of differentially expressed genes involved in cell proliferation in the zebrafish fast muscle in response to exercise. (PDF 23 KB) [file 12864_2014_6880_MOESM6_ESM.pdf]

Table S7. Canonical pathways that were significantly altered (Fisher's exact test, $p < 0.05$) in zebrafish fast muscle in response to swimming. The number of differentially expressed genes in relation to the total number of genes present in each pathway in the Ingenuity Knowledge Base (No. Genes) and their identity (Pathway molecules) are shown.

**Ingenuity Canonical Pathways**	**p-value**	**No. Genes**	**Pathway molecules**
Integrin Signaling	3,28E-16	94/208	RAP2B,MAP2K4,RAC2,RAF1,MAPK1,MYL2,ARPC5, TLN1,PIK3R4,PTEN,NCK2,PAK1,RHOG,MAP2K2,RHOB,PIK3CG,ARF4,MAPK3,CAV1,ITGB4,ACTG2,TSPAN4, CAPN5,RND2,ACTR2,PIK3C2B,TSPAN5,RRAS,CRKL, RAC1,TSPAN2,RHOJ,RAP1A,RAC3,TTN,MYL9,CAPN8, ARF5,ARPC1A,MYL12B,ARF3,RHOA,ARPC2,ZYX, PPP1R12A,CAPN2,PAK7,ACTN4,CTTN,FNBP1,CAPN3, RAP1B,FYN,ITGA2B,TSPAN7,RHOT2,ACTA2,PIK3R5, Actn3,ILK,CRK,MYLK,CDC42,RHOH,BRAF,ARF6, ACTR3,WASL,RHOU,ARPC3,RHOF,ACTC1,MAP2K1, VASP,CAPN10,ITGB1,PXN,PAK2,NRAS,PIK3C2A,GRB2,ARPC5L,ASAP1,RHOC,ACTB,MAPK8,Arf1,PLCG1, ACTG1,ITGB2,ARF1,CAPNS1,LIMS1,ARPC4
Protein Ubiquitination Pathway	3,62E-12	103/268	CRYAB,BAG1,USP10,PSMA2,PSMC2,DNAJB12,PSMD5, THOP1,HSPA8,TRAF6,PSMB2,PSMD12,PSMA5,PSMB1, PSMA4,DNAJC5B,PSMD1,HSPB7,SMURF2,UBE2C, ANAPC2,PSMD7,PSMD9,CUL1,DNAJC13,CDC23, DNAJA1,USP39,PSMC6,PSMD14,AMFR,UCHL3,MED20, PSMD13,MDM2,USP33,PSMD8,DNAJC11,USP44,PSMC1,PSME1,USP4,USP49,PSMA3,HSPA14,USP5,UBE2D2, HSPA5,PSMC5,HSPA4,USP7,USP8,UBE2B,DNAJC8, UCHL5,USP40,NEDD4L,PSMA6,PSMB5,UBE4B,USP36, HSPA9,BIRC6,PSMC4,PSMD6,TCEB2,PSMD3,ANAPC4, PSMD11,UBE2L3,DNAJC5,UBE2H,UBE2G1,DNAJC18, PSMD4,UBE2E1,UBE2I,PSMB3,B2M,USP14,DNAJC12, DNAJC10,PSMB6,PSMD10,HSPA12B,DNAJC4,HSPE1, UBE2V1,PSMB4,PSMA1,HSPD1,DNAJB9,UBE2D1,XIAP,DNAJC21,NEDD4,DNAJB11,PSMD2,UBA1,CDC34,UBC,PSMC3,DNAJC7
Wnt/β-catenin Signaling	5,57E-11	75/175	WNT10B,CSNK1G1,FZD3,PPP2CA,WNT16,GSK3A,NT6, CCND1,TCF7,MYC,SOX2,WIF1,TGFB1,WNT7B,RARA,PM1L,CSNK2A1,WNT4,RUVBL2,FZD2,TP53,AXIN2, CSNK1G2,CREBBP,FZD9,ACVR1B,APC,PPP2CB,PP2R1A,CDH2,PPP2R4,GNAO1,TLE3,FZD5,SFRP1,DVL2, WNT1,FZD10,PPP2R2A,MARK2,ILK,ACVR2B, KREMEN1,WNT2,EP300,SOX17,CSNK1E,WNT7A,NLK, RARB,SMO,TGFB2,SFRP5,PPP2R5C,CTNNB1,SOX5, PPARD,DVL1,HDAC1,GNAQ,MDM2,TCF7L1,CSNK2A2,FZD8,WNT8A,FZD4,WNT10A,NR5A2,SOX9,DVL3, DKK1,UBC,WNT11,LRP1,TCF7L2
mTOR Signaling	6,06E-06	68/211	MAPK1,PRKAB1,PPP2CA,EIF4A2,PIK3R4,RPS7,RHOG, RHOB,EIF4G2,EIF3D,MAPK3,PIK3CG,INS,PPM1L, PRKAA1,RPS10,EIF4B,RND2,PIK3C2B,RRAS,RAC1, VEGFC,RHOJ,EIF3E,MLST8,EIF3M,ATG13,PPP2CB, PPP2R1A,RPS4X,PPP2R4,RHOA,IRS1,EIF4A3,PRKCD, RPS6KA1,FNBP1,ULK1,PPP2R2A,RHOT2,PIK3R5, FKBP1A,EIF4G1,RHOH,EIF4E,PRKAG1,EIF4EBP1, RPS27,EIF3A,RHOU,PRKCE,PPP2R5C,RHOF,RPS24, NRAS,EIF3H,PIK3C2A,RHOC,RPS2,EIF3J,PLD4,EIF3G, RPS26,EIF3I,PRKAG2,PRR5,EIF3L,RPS14
TGF-β Signaling	7,22E-06	36/89	MAP2K4,RAF1,NODAL,BMP4,MAPK1,HOXC9,MAPK13

			,ACVR2B,BMPR1B,TLX2,TGIF1,EP300,MAP2K2, RUNX2,BMPR1A,TGFB1,MAPK3,TGFB2,TFE3,MAP2K1 ,SMAD2,NRAS,SMAD9,GRB2,RRAS,HDAC1,CREBBP, MAPK8,SMAD7,SMAD6,ACVR1B,INHBB,TRAF6, MAPK14,PIAS4,SMURF2
Ephrin B Signaling	2,46E-05	32/82	RAC2,ITSN1,MAPK1,GNA11,GNB5,CXCL12,CDC42, HNRNPK,GNG7,LIMK1,ROCK2,NCK2,GNB1,EFNB2, PAK1,MAPK3,ITSN2,EFNB3,GNA13,CTNNB1,EPHB4, PXN,KALRN,RAC1,GNAI1,GNAQ,RAC3,GNG10,RHOA, ACP1,GNAO1,GNG5
Actin Cytoskeleton Signaling	3,04E-05	72/239	RAF1,RAC2,MAPK1,MYL2,ARPC5,TLN1,PIK3R4,SSH1, F2,LIMK1,ROCK2,PAK1,CYFIP2,MAP2K2,FGF18, PIK3CG,EZR,MAPK3,INS,ACTG2,GNA13,PIK3C2B, ACTR2,TIAM1,RRAS,CRKL,RAC1,GSN,TTN,RAC3,APC ,MYL9,ARPC1A,MYL12B,RHOA,ARPC2,ARHGAP35, PPP1R12A,PAK7,ACTN4,PIP4K2C,ABI2,PDGFA,ACTA2 ,Actn3,PIK3R5,TRIO,CRK,MYLK,MYH11,CDC42,FGF13, WASL,ACTR3,ARPC3,ACTC1,MAP2K1,NCKAP1,ITGB1 ,PXN,NRAS,PAK2,PIK3C2A,GRB2,ARPC5L,CSK, MYLPF,ACTB,ACTG1,FGF20,ARPC4,MSN
IGF-1 Signaling	6,53E-05	38/105	RAF1,MAPK1,YWHAH,PIK3R5,SRF,IGFBP7,PIK3R4, PRKAG1,CASP9,MAP2K2,PIK3CG,MAPK3,FOXO3, CSNK2A1,IGF1R,IRS2,IGFBP1,MAP2K1,PIK3C2B,PXN, YWHAG,NRAS,PIK3C2A,YWHAE,GRB2,RRAS, YWHAB,MAPK8,YWHAZ,CSNK2A2,NEDD4,PTPN11, IRS1,PRKACG,IGFBP3,PRKACA,PRKAG2,CYR61
Glycolysis I	1,01E-04	14/41	PGK1,PKM,GAPDH,PFKP,BPGM,PFKM,ENO1,GPI,TPI1, PGAM1,ALDOA,FBP1,GAPDHS,ALDOC
VEGF Signaling	1,03E-04	36/104	EIF1AY,RAF1,EIF2B4,MAPK1,ACTA2,PIK3R5,Actn3, EIF2S1,PIK3R4,ELAVL1,ROCK2,MAP2K2,EIF1AX, MAPK3,PIK3CG,FOXO3,ACTG2,MAP2K1,ACTC1, PIK3C2B,PTPN6,PXN,NRAS,YWHAE,PIK3C2A,GRB2, RRAS,ACTB,VEGFC,PLCG1,ACTG1,EIF2S2,PTPN11, EIF2B1,EIF2B5,ACTN4
AMPK Signaling	4,67E-04	46/169	ADRA2B,CAB39,PRKAB1,PFKFB1,MAPK1,PPP2CA, PPP2R2A,PIK3R5,SMARCD2,MAPK13,PIK3R4, SMARCA4,PRKAG1,EIF4EBP1,PPM1D,AK1,PIK3CG, PPM1L,INS,PRKAA1,PPM1A,IRS2,PPP2R5C,ADRB2, TAF9,PIK3C2B,ACACB,CPT1A,CKM,PIK3C2A,AK3, PFKP,PPM1G,PFKM,PPP2CB,PPP2R1A,MAPK14, PPP2R4,IRS1,PRKACG,CPT2,PRKACA,PRKAG2,AK2, HLTF,ADRA1A
Calcium Signaling	6,03E-04	58/213	RAP2B,MYL2,MAPK1,ATP2B1,NFATC3,Tpm1,TPM1, GRIA4,ATP2A2,HDAC6,CAMK2A,CAMK2D,MAPK3, TNNT3,PPP3R1,CASQ1,TPM4,HDAC4,CREBBP,SLC8A2 ,CREB3L4,PPP3CC,RAP1A,MYL9,HDAC3,PRKACG, PRKACA,RAP1B,CAMK4,TNNI2,GRIA1,ACTA2,MYH11 ,PRKAG1,NFATC1,GRINA,EP300,ATP2A1,NFAT5, CREB1,CAMK1G,RYR1,ACTC1,PPP3CA,CALR,GRIN1, LETM1,TP63,HDAC1,MEF2A,MCU,NFATC4,ATP2B2, CALM1,MEF2D,PRKAG2,MEF2C,GRIA3
Insulin Receptor Signaling	1,22E-03	44/142	RAF1,FYN,EIF2B4,ASIC2,MAPK1,SGK1,PPP1R3C, PIK3R5,CRK,GSK3A,PIK3R4,PPP1R14B,EIF4E,PRKAG1 ,EIF4EBP1,PTEN,MAP2K2,PPP1R7,MAPK3,PIK3CG, PTPN1,FOXO3,INS,IRS2,STX4,PPP1CA,MAP2K1, PIK3C2B,NRAS,PIK3C2A,GRB2,RRAS,CRKL,MAPK8, PPP1R14A,PTPN11,GAB1,IRS1,PRKACG,EIF2B1,EIF2B5 ,PRKACA,PRKAG2,PPP1R12A
FGF Signaling	1,53E-03	31/92	RAF1,MAPK1,PIK3R5,CRK,MAP3K5,MAPK13,PIK3R4, EP300,FGF13,FGF18,MAPK3,PIK3CG,CREB1,MAP2K1, PIK3C2B,PTPN6,PIK3C2A,GRB2,CRKL,FGFR1,CREBBP ,MAPK8,RAC1,FGFR2,PLCG1,CREB3L4,MAPK14,

			PTPN11,GAB1,FGF20,FGFRL1
Chemokine Signaling	1,72E-03	26/73	RAF1,NRAS,CAMK4,MYL2,MAPK1,RRAS,GNAI1, MAPK8,GNAQ,CXCL12,PLCG1,MAPK13,LIMK1, ROCK2,CALM1,MAPK14,CAMK2D,CAMK2A,MAP2K2, RHOA,MAPK3,PIK3CG,CAMK1G,PPP1R12A,MAP2K1, OPN1SW
PI3K/AKT Signaling	1,75E-03	41/144	RAF1,GAB2,RELA,MAPK1,YWHAH,PPP2CA,PPP2R2A, ILK,GSK3A,MAP3K5,CCND1,EIF4E,EIF4EBP1,PTEN, IKBKB,IKBKG,MAP2K2,MAPK3,PIK3CG,FOXO3, PPM1L,PPP2R5C,CHUK,CTNNB1,MAP2K1,MCL1, ITGB1,TP53,NRAS,YWHAG,YWHAE,GRB2,RRAS, YWHAB,YWHAZ,MDM2,PPP2CB,PPP2R1A,GAB1, LIMS1,PPP2R4
Fatty Acid β-oxidation I	1,99E-03	14/45	ACSL3,ECHS1,SLC27A2,ACSL6,ACSBG2,ACAA2,ECI1, HADHB,ECI2,ACSL4,SLC27A1,ACADM,HADH,HADHA
Hypoxia Signaling in the Cardiovascular System	4,35E-03	24/67	TP53,P4HB,COPS5,CREBBP,UBE2D2,NQO1,BIRC6, MDM2,CREB3L4,UBE2D1,PTEN,EP300,UBE2L3,UBE2H ,EDN1,UBE2B,UBE2G1,CREB1,UBE2V1,HIF1AN, CDC34,UBE2E1,UBE2C,UBE2I
PDGF Signaling	4,63E-03	27/85	MAP2K4,RAF1,MAPK1,PDGFA,PIK3R5,SRF,CRK, PIK3R4,MYC,MAP2K2,MAPK3,PIK3CG,CSNK2A1, CAV1,CAV3,MAP2K1,PIK3C2B,NRAS,PIK3C2A,RRAS, GRB2,CRKL,MAPK8,PLCG1,CSNK2A2,ABL2,ACP1
HIF1α Signaling	9,07E-03	33/108	NOS1,MAPK1,MMP14,MAPK15,PIK3R5,MAPK13, NAA10,PIK3R4,LDHB,EP300,EDN1,SLC2A2,PIK3CG, MAPK3,NOS2,APEX1,TP53,PIK3C2B,NRAS,PIK3C2A, RRAS,MAPK4,COPS5,CREBBP,MAPK8,MAPK6,VEGFC ,MMP10,MDM2,TCEB2,MAPK14,EGLN1,MAPK7
Notch Signaling	9,60E-03	15/43	FURIN,DLL1,JAG2,HES5,HES1,DTX2,NUMBL,NOTCH2 ,MAG,LFNG,HEY2,JAG1,NOTCH1,HEY1,PSEN1
Angiopoietin Signaling	3,00E-02	21/74	RELA,PIK3C2B,ANGPT2,PAK2,NRAS,PIK3C2A,GRB2, RRAS,PIK3R5,CRK,PIK3R4,IKBKB,IKBKG,TNIP1,PAK1 ,CASP9,PTPN11,PIK3CG,PAK7,CHUK,STAT5B
