# Supplementary material for: Swimming-induced exercise promotes hypertrophy and vascularization of fast skeletal muscle fibres and activation of myogenic and angiogenic transcriptional programs in adult zebrafish
Source: BMC Genomics. 2014 Dec 18;15(1):1136. doi: 10.1186/1471-2164-15-1136 (PMC4378002; doi:10.1186/1471-2164-15-1136)
Supplement: Supplementary file 7 — Additional file 7: Table S7: Canonical pathways that were significantly altered (Fisher’s exact test, p < 0.05) in zebrafish fast muscle in response to swimming. The number of differentially expressed genes in relation to the total number of genes present in each pathway in the Ingenuity Knowledge Base (No. Genes) and their identity (Pathway molecules) are shown. (PDF 5 KB) [file 12864_2014_6880_MOESM7_ESM.pdf]

**Table S8.** Quantitative real-time PCR (qPCR) validation of microarray results from selected genes

| Ensmbl ID | Gene name | Regulation | Microarray | qPCR |
|---|---|---|---|---|
| ENSG00000164434 | FABP7 | Down-regulated | -3,96 | -2,31 |
| ENSDARG00000055216 | TUBA1B | Down-regulated | -4,13 | -3,20 |
| ENSDARG00000012234 | PSME3 | Down-regulated | -3,11 | -3,16 |
| ENSDARG00000003526 | PSMA5 | Down-regulated | -2,43 | -2,40 |
| ENSDARG00000013804 | CAPNS1 | Down-regulated | -1,29 | 1,39 |
| ENSG00000127418 | FGFRL1 | Up-regulated | 4,32 | 1,81 |
| ENSDARG00000019150 | FOXA1 | Up-regulated | 4,22 | 2,71 |

Microarray analysis was succesfully validated by qPCR. Results showed the same regulation trend for both techniques. Data shown represent mean fold change and real-time PCR data are shown as the mean ± S.E.M. normalized to RPS15 as a reference gene.
