# Supplementary material for: Swimming-induced exercise promotes hypertrophy and vascularization of fast skeletal muscle fibres and activation of myogenic and angiogenic transcriptional programs in adult zebrafish
Source: BMC Genomics. 2014 Dec 18;15(1):1136. doi: 10.1186/1471-2164-15-1136 (PMC4378002; doi:10.1186/1471-2164-15-1136)

Figure S1. IPA-based network generated from molecules involved in cell proliferation that are differentially expressed in fast muscle of exercised adult zebrafish. The shapes of the genes correlate with the functional classification symbolised in the legend. Arrows represent the direct relationship between molecules. Color intensity correlates to transcription value, calculated as log2ratio (exercised/non-exercised); green represents molecules with repressed transcription (negative log2ratio); red represents molecules with enhanced transcription (positive log2ratio).

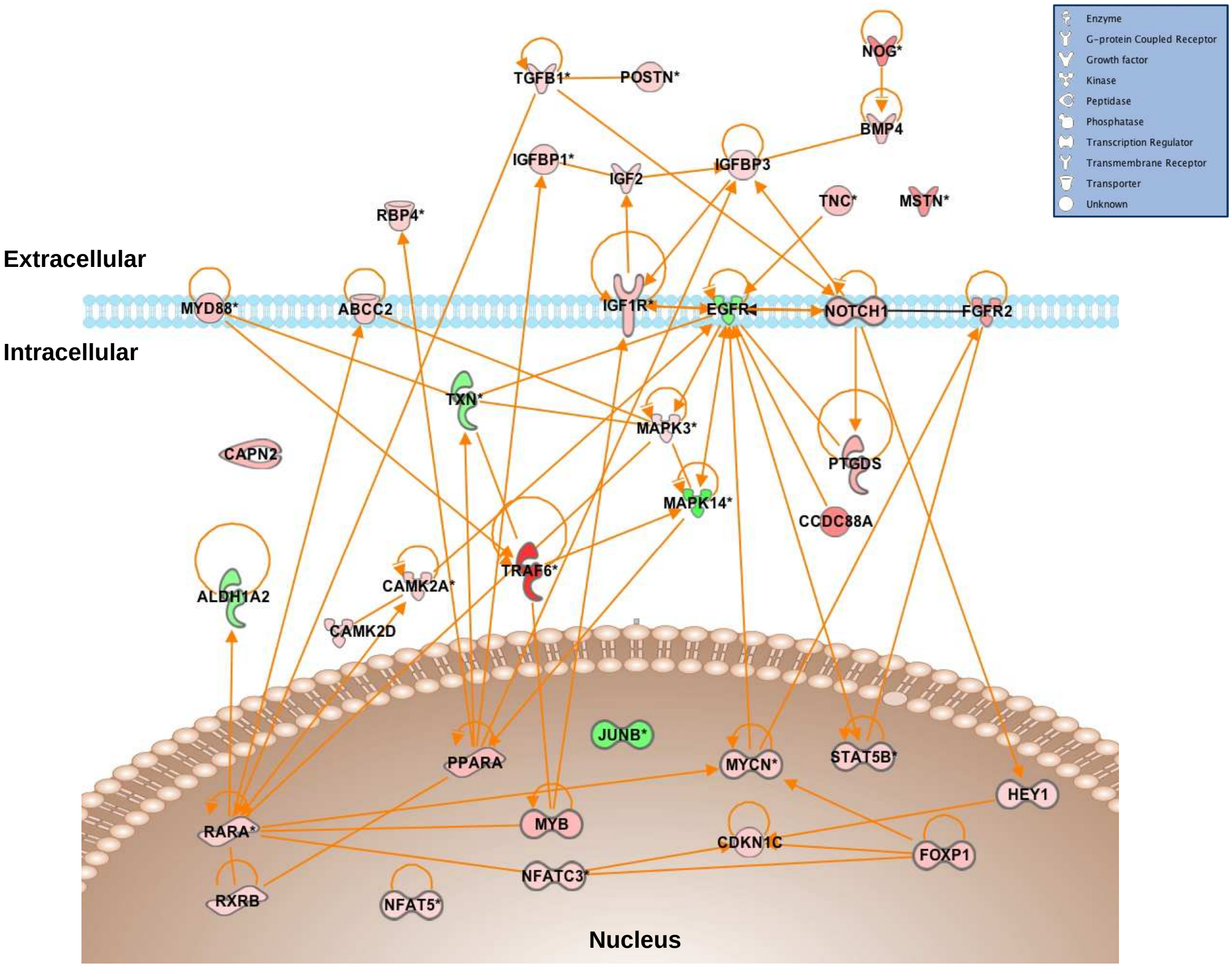

Supplement: Supplementary file 8 — Additional file 8: Table S8: Quantitative real-time PCR (qPCR) validation of microarray results from selected genes. (PDF 360 KB) [file 12864_2014_6880_MOESM8_ESM.pdf]
