# Supplementary material for: Swimming-induced exercise promotes hypertrophy and vascularization of fast skeletal muscle fibres and activation of myogenic and angiogenic transcriptional programs in adult zebrafish
Source: BMC Genomics. 2014 Dec 18;15(1):1136. doi: 10.1186/1471-2164-15-1136 (PMC4378002; doi:10.1186/1471-2164-15-1136)
Supplement: Supplementary file 9 — Additional file 9: Figure S1: IPA-based network generated from molecules involved in cell proliferation that are differentially expressed in fast muscle of exercised adult zebrafish. The shapes of the genes correlate with the functional classification symbolised in the legend. Arrows represent the direct relationship between molecules. Color intensity correlates to transcription value, calculated as log2ratio (exercised/non-exercised); green represents molecules with repressed transcription (negative log2ratio); red represents molecules with enhanced transcription (positive log2ratio). (PDF 6 KB) [file 12864_2014_6880_MOESM9_ESM.pdf]

Table S9. Sequences of primers used in gene expression analyses by qPCR

Ensmbl ID	Gene name	Primer sequences (5'- 3')	Amplicon size (bp)
ENSG00000164434	FABP7	(F)TGATGAAACCACAGCAGACGATAG	202
		(R)CCTGCGCTCTGGACATTATGC	
ENSDARG00000055216	TUBA1B	(F)GGTATGGAGGAGGGCGAGTTCT	139
		(R)GGAAACACAGCAGGCAGCATT	
ENSDARG00000012234	PSME3	(F)GATGGAGCAGGAGACGACAAAGTT	252
		(R)GCTTCCCCTTCCACTGTTCTGA	
ENSDARG00000003526	PSMA5	(F)CAATGAGACGATGACTGTGGAGAG	196
		(R)TCTCGCGTCACACTGGACAAA	
ENSDARG00000013804	CAPNS1	(F)CGGTACAATCAAGGTCAATGTTCA	227
		(R)CGTAGTGAGAAACCGTCCCAGTG	
ENSG00000127418	FGFRL1	(F)ACCAGCATTTCCATTACCAGTGTT	141
		(R)CGAGGTCCATCAGAACTGCGAT	
ENSDARG00000019150	FOXA1	(F)ACCCTTCGGCGGCTTTAGAG	82
		(R)CGTGGCAATGACTACGATGTATTG	
ENSDARG00000070849	RPS15	(F)CTCAGGAGGAAGCAGCAGTCTCT	106
		(R)CCATGTCTCTCAGGTGAGTTTTGAC	
